# Cutaneous T-Cell Lymphoma: Yin-Yang Effects of Transcription Factors HLF and NFIL3 in Regulation of Malignant T-Cell Markers in the Context of HDAC Inhibitor Romidepsin Treatment

**DOI:** 10.3390/cancers17142380

**Published:** 2025-07-17

**Authors:** Andrew V. Kossenkov, Noor Dawany, Sonali Majumdar, Celia Chang, Calen Nichols, Maria Wysocka, Richard Piekarz, Michael K. Showe, Susan E. Bates, Alain H. Rook, Ellen J. Kim, Louise C. Showe

**Affiliations:** 1 The Wistar Institute, 3601 Spruce Street, Philadelphia, PA 19104, USA; 2 Department of Dermatology, University of Pennsylvania, Philadelphia, PA 19104, USA; 3 Cancer Therapy Evaluation Program, National Cancer Institute, Rockville, MD 20850, USA; 4 Department of Hematology/Oncology, Columbia University Irving Medical Center, New York, NY 10032, USA; 5 Department of Hematology/Oncology, James J. Peters VA Medical Center, Bronx, NY 10468, USA

**Keywords:** CTCL, HDAC inhibitor, romidepsin, PAR bZIP

## Abstract

Background/Objectives: We examined the in vivo effects of successive treatments with the histone deacetylase (HDAC) inhibitor romidepsin in patients with cutaneous T-cell lymphoma (CTCL), using changes in gene expression in peripheral blood mononuclear cells (PBMCs). Methods: Exploiting data from a highly responsive CTCL patient through 12 months of treatment, we identified a malignant cell predictor (MCP), a gene signature associated with the diminishing numbers of circulating malignant cells. Results: The MCP was successfully validated in the patient’s relapse sample 9 months after treatment was terminated and via an independent set of CTCL patient samples. Conclusions: The MCP set of genes contained novel CTCL markers, including membrane-associated proteins not normally expressed in lymphocytes. A subclass of those markers was also detectable in residual malignant cells undetected by flow cytometry in remission samples from a patient who relapsed 10 months later. We identified a subset of transcriptional regulators, miRNAs and methylation patterns associated with the effect of progressive treatments revealing potential mechanisms of transcriptional dysregulation and functional effects in the malignant cells. We demonstrate a role for transcriptional activator HLF, over-expressed in malignant cells, and downregulated transcriptional-suppressor and immune-modulator NFIL3, as regulators of CTCL-specific genes.

## Simple Summary

The drug romidepsin, which is known to regulate gene expression, has been shown to be an effective treatment in a subset of patients with cutaneous T-cell lymphoma (CTCL). We examined gene expression changes that occurred through repeated romidepsin treatments using carefully collected blood cell samples from treated CTCL patients. Focusing on data from a highly responsive CTCL patient through 12 months of treatment, including a period free from disease, we identified new markers that are characteristic of the malignant cells and difficult-to-detect residual disease. We identified effected cell functions and potential mechanisms of dysregulation through changes in miRNA expression and DNA methylation patterns. We also found a significant impact of transcriptional regulators HLF (activator) and NFIL3 (suppressor) that control the expression of malignant-specific genes. We report new surface markers for the malignant cells, including markers identifiable in residual disease, and new potential therapeutic targets.

## 1. Introduction

Cutaneous T-cell lymphomas (CTCL) are a heterogeneous group of non-Hodgkin lymphomas with characteristics of “skin-homing” T lymphocytes that primarily affect older individuals. The most common CTCL forms include the “indolent” skin associated mycosis fungoides (MF) and Sézary syndrome (SS), an aggressive erythrodermic and leukemic variant with poor prognosis. CTCL cells are overwhelmingly CD45^+^RA^+^CD4^+^Th2-like T cells, with hallmarks that include a highly characteristic cerebriform nucleus [1], the loss of CD26 (di-peptidyl peptidase 4/DPP4) surface expression [2,3], and the additional loss of CD7 expression in some cases [4,5]. Because CD4 cells that lack CD26 surface expression or have cerebriform nuclei are also normally present at low but variable numbers in older unaffected individuals, it is difficult to identify the small numbers of circulating malignant cells present in early disease or the small numbers of residual malignant cells that may persist early in recurrence after apparently successful treatments. Our previous gene expression profiles of the circulating Sézary T cells identified a number of important malignant cell markers including a significant downregulation of key Th1 transcription factor STAT4, an increase in the central Th2 transcription factor GATA3, the increased expression of Jun B, and the unique expression of tissue plastin (PLS3) [6], a gene not normally expressed in lymphoid cells. PLS3 is a single gene marker of the malignant cell, and detection of the mRNA by PCR [7] or its protein by immunohistochemistry [8] is informative for approximately 75–80% of SS patients [6,8].

In general, chemotherapy has proven to have little effect on the long-term survival of Sézary patients, although biological response modifiers such as IFNα plus photopheresis prolong survival in a subset of patients [9,10,11]. Histone deacetylation (HDAC) inhibitors including romidepsin (Istodax) and SAHA (vorinostat, Zolenza) have been shown to be particularly effective in the treatment of CTCL, suggesting a strong epigenetic component in its etiology [12,13,14,15,16].

Epigenetic regulation of gene expression through histone acetylation and deacetylation alters the transcription of the genes involved in a variety of different processes including cell growth, maturation, survival, and apoptosis. HDAC inhibitors have demonstrated the potential to be potent anticancer agents and are noted for their ability to induce tumor-suppressor genes as well as genes involved in cellular differentiation and apoptosis frequently found to be repressed in malignant cells. A number of HDAC inhibitors have been tested in clinical trials to determine the anti-cancer efficacy of these agents on solid tumors, with varying results [17] but with encouraging results in the treatment of hematopoietic cancers and in particular the TCLs including CTCL [18]. While a principal effect of the HDAC inhibitors, including romidepsin, is to stimulate apoptosis in the malignant cells [19,20], the mechanism of action in inducing tumor cell death is not completely understood. Recent studies have noted that histone acetylation depletes acetyl CoA pools, resulting in metabolic stress in vulnerable cells [21,22]. Many downstream effects of HDAC inhibition have been described, and it is not entirely clear which are due to gene expression changes, which are due to ensuing chromatin disruption, and which are due to the metabolic impacts of acetyl CoA depletion [23]. Such impacts include disruption of co-repressor complexes, induction of oxidative injury, upregulation of the expression of death receptors, generation of lipid second messengers such as ceramide, interference with the function of chaperone proteins, and modulation of the activity of NFkB as critical determinants of lethality [24].

While encouraging overall response rates were reported for multi-center phase II trials with romidepsin [25,26,27] and SAHA [28,29,30], a significant number of patients did not respond to therapy and others relapsed after ceasing treatment. Due to possible delays of HDAC inhibitor’s effects and because of the potential importance of the immune system in this response, studies of cell lines or even isolated malignant cells in vitro give an incomplete picture of what is happening in vivo. To understand the parameters that define response and resistance to romidepsin treatment in vivo, we systematically analyzed protein-coding and miRNA expression as well as DNA methylation patterns through successive treatments in 5 CTCL patients that were part of an international phase II clinical trial. An overall clinical assessment was reported in Whittaker et al. [27]. This study included one complete responder, three partial responders, and one patient classified as a non-responder with treatments ranging from 2 to 12 months.

## 2. Materials and Methods

### 2.1. Study Populations

Samples were collected from 5 cutaneous T-cell lymphoma (CTCL) patients recruited at the Hospital of the University of Pennsylvania (HUP) as part of a multi-site phase II trial to confirm the efficacy of romidepsin treatment in patients with refractory CTCL. Enrolled patients ranged from stage IB to IVA CTCL and had failed one or more prior systemic therapies. Staging was based on the original American Joint Committee on Cancer (AJCC) criteria according to the Tumor-Node-Metastasis-Blood (TNMB) categories and the overall staging system [31]. Informed consent forms were approved by the appropriate institutional review boards before patients were enrolled. All patients provided written informed consent to participate in the study before any study-specific procedures were performed.

**Figure 1:**
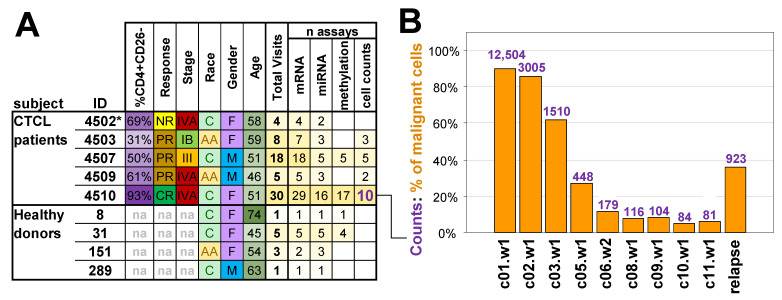
Study population and malignant cell counts. (**A**) Information and data collected from subjects. IB/III/IVA = overall stage from the tumour-node-metastasis-blood (TNMB) classification, na = not applicable, NR = non-responder, PR = partial responder, CR = complete responder, C = Caucasian, AA = African American, F = female, M = male, * the clinical assessment of patient p4502 as NR was based on a very short treatment regimen. (**B**) Changes in the calculated proportion of malignant cells in assayed samples during the treatment course for complete responder patient p4510. Bars indicate absolute counts of malignant CD4^+^CD26^−^ cells by flow cytometry expressed as cells per ul. The relapse sample was collected 10 months after the final cycle 12 treatment. Time points are represented by treatment cycle number (c) followed by week number (w).

The clinical protocol is described in detail in Whittaker et al. [27]. The 5 patients enrolled in the HUP arm of the study were diagnosed with Sézary syndrome, the leukemic form of CTCL. The protocol in brief is as follows: patients received romidepsin 14 mg/m^2^ as a 4 h intravenous infusion on days 1, 8, and 15 (week 1, week 2, and week 3) of each 28-day cycle for six cycles. The summary of the patient demographics and their time on therapy are shown in Figure 1A. Patients with stable disease or measurable response could opt for an extension of treatment beyond 6 cycles as was the case with patient p4510 [32] included in this study. Additional samples from patients with Sézary syndrome, including data from purified monoclonal CD3^+^CD4^+^ cells [33], used in the study were provided by Drs. Alain Rook (UPenn) and Susan Bates (then at NCI). Normal controls matched by gender, race, and age were collected through the Wistar Phlebotomy Facility during the same morning time period that patient samples were collected. The collection and analysis of all samples were carried out with appropriate IRB approval.

### 2.2. Clinical Response

Disease assessment was conducted at screening, baseline, the first day of all subsequent cycles, and 30 days after the last cycle. A complete response was defined as a complete resolution of skin patches, plaques, and tumors (or erythroderma); no evidence of abnormal lymph nodes; complete clearing of skin disease with confirmation by skin biopsy; and the absence of circulating Sézary cells. A detailed case study of the patient who was diagnosed as having a complete response is described in Kim et al. [32]. A partial response was defined as a ≥50% improvement in skin, lymph node, and in peripheral blood; no worsening in lymph nodes or circulating Sezary cells; and no evidence of new tumors (cutaneous or non-cutaneous). CR/PR documentation required confirmation of response after 4 weeks [32].

### 2.3. Clinical Assessment of Circulating Tumor Burden

The presence of circulating malignant Sézary cells in the peripheral blood was assessed by flow cytometry in the Wistar Flow Cytometry Facility and defined as the percent of lymphocytes that were CD4^+^/CD26^−^, as reviewed in [34]. Due to repeated weekly sample collections, collected sample volumes were restricted, eliminating the possibility of purifying malignant cells from each treatment. Those visits during which malignant cell levels were also assessed by FACS are indicated in Supplementary Table S1. Response to treatment was determined, in part, by the assessment of changes in erythroderma, a reduction in the CD4:CD8 cell ratio, and a decrease in circulating CD4^+^CD26^−^ cells as assessed by flow cytometry [32]. All 5 patients that entered the HUP arm of the trial, regardless of their time on romidepsin therapy, were included in the gene expression studies.

### 2.4. PBMC Collection and Processing

Peripheral blood samples were collected into CPT tubes (BD) at baseline and before each romidepsin intravenous infusion was started on days 1, 8, and 15 of every 28-day treatment cycle. Samples from healthy donors were also collected in the morning, and a subset of samples were collected at weekly intervals of 1 to 3 weeks to match the collection protocols for patients, as indicated in Supplementary Table S1. CPT tubes were processed for PBMC isolation, within 1 h of collection. RNA purification was carried out using TRI Reagent^®^ (Molecular Research Center, Cincinnati, OH, USA), as recommended, and controlled for quality with the Bioanalyzer (Agilent, Santa Clara, CA, USA). Only samples with RIN numbers of 7.0 or greater were used for further studies. DNA was collected from the Tri-reagent interphase of the same samples according to the manufacturer directions.

### 2.5. mRNA, miRNA, and Methylation Studies

mRNA expression was assayed on Illumina WG-6v2 human whole-genome bead arrays using a constant amount (400 ng) of total RNA. Illumina BeadStudio v.3.0 software was used to export expression levels and detection *p*-values for each probe of each sample. Arrays were quantile-normalized and filtered to remove non-informative probes. A probe was regarded as non-informative if it had a detection *p*-value > 0.05 in all samples or if the maximum ratio between expression values was below 1.2 between all sample pairs. miRNA analysis was carried out on the Illumina MicroRNA v2 Sentrix Beadchip arrays using 500 ng of the same RNA. Data was quantile-normalized and filtered to include only those miRNAs that were significantly expressed in at least one sample. The DNA for methylation studies was isolated from the Tri-reagent interphase, processed as recommended by Illumina (San Diego, CA, USA), and analyzed on the Illumina 27 k human methylation array. Methylation data are presented as beta values representing the ratio of the methylated probe relative to the total methylated and unmethylated levels. The beta value ranges from 0 to 1, corresponding to the completely unmethylated and methylated sites, respectively. RNA-seq was carried out on Tru-seq or Script-seq libraries using 75 bp and 36 bp single-end GAIIX run and paired-end HiSeq runs. The mRNA 150 bp paired ends were analyzed on the Next-Seq 500 platform. Raw RNA-seq reads were aligned to the GRCh37 reference human genome using TopHat (v2.0.3) [35] and mapped reads were then assembled to transcripts using Cufflinks (v2.0.0) [36]. Assemblies were merged across all sequenced samples using Cuffmerge (v2.0.0) [36], and FPKM values were used for downstream analyses.

### 2.6. Quantitative Real-Time PCR

qRT-PCR validation of array results was carried out using the ABI TaqMan system (Applied Biosystems, Waltham, MA, USA) as recommended, in an ABI 7900HT PCR System. Each sample was analyzed in triplicate, and samples with an absolute difference in Ct values between replicates of more than 0.5 Ct were repeated. GAPDH was used as an endogenous control, and absolute expression values were calculated as 2^CtGAPDH−Ct^.

### 2.7. Flow Cytometry

For flow cytometry experiments, 10–12 M of cells were thawed and washed in PBS to remove freezing medium containing DMSO centrifuged at 1500 rpm for 5 min at 4 °C, and the supernatant was discarded. The pellet was re-suspended in FACS buffer (PBS supplemented with 10% fetal bovine serum (FBS)), and 1–2 M of cells were added to 3 tubes and incubated with previously optimized dilutions of directly conjugated fluorescently labeled antibodies verified for FACS. All 3 tubes were incubated with anti-CD4 and anti-CD26 and either anti-CD164, PD1, or MUC1. Appropriate IgG controls were assayed in parallel in separate tubes of cells. Incubation was carried out in the dark on ice for 1 h. Cells were then washed 3× in FACS buffer. Live/dead staining was performed to exclude dead cells using Invitrogen (Carlsbad, CA, USA) Live/Dead Fixable Dead cell stain kit (Aqua; L/D). Stained cells were assayed on the FACS LSRII flow cytometer (Becton Dickinson, San Jose, CA, USA) according to manufacturer’s instructions. The analysis was completed using FlowJo software (version 10.7, Tree Star, Ashland, OR, USA). The antibodies used and the manufacturers they were purchased from were as follows: CD4 (fluorochrome: PE/Cy7; clone: OKT4) and CD26 (fluorochrome: PE; clone: BA5b) from BioLegend (San Diego, CA, USA), MUC1/CD277 (fluorochrome: Alexa-fluor 488; clone: SM3) and PD1/CD279 (fluorochrome: APC; clone: eBioJ105) from eBioscience (San Diego, CA, USA), and CD164 (fluorochrome: PerCP/Cy5.5; clone: N6B6) from BD Pharmingen (Becton Dickinson, San Jose, CA, USA). 

### 2.8. Calculation of the Proportion of Malignant Cells in a Sample

Calculation of the proportion of malignant cells was performed for samples that had the numbers of monocytes and lymphocytes counted manually by microscopy, and the numbers of T cells (T = CD3^+^ marker), B cells (B = CD3^−^ CD20/22^+^ marker), NK cells (NK = CD5^−^ CD56/16^+^ marker), and malignant cells (Tm = CD4^+^CD7^−^CD26^−^ marker) was determined by flow cytometry.

### 2.9. Detection of Genes Over-Expressed in Malignant Cells

With only p4510 samples, a multivariate regression model was used to correlate the expression data of a gene with the proportion of malignant cells present, with potential batch effects being accounted for as described in detail in the Supplementary Methods. The significance of the correlation was corrected for multiple testing using the Benjamini–Hochberg procedure, and the cutoff was set at FDR < 5%. The significant genes were further restricted to those whose expression in the final 9–12 cycles did not show significantly lower levels (*p* > 0.5) compared to the expression of the gene in the healthy donor samples. Genes satisfying these two criteria were considered to be malignancy-specific.

### 2.10. Prediction of the Proportion of Malignant Cells Present in a Sample

Expression data from the 1479 malignancy-specific genes for patient p4510 was used as a training set to develop a malignant cell predictor (MCP) as described in detail in the Supplementary Methods. The parameters obtained from the training set were then used to calculate a predicted percent of malignant cells for every sample, including the training set samples (n = 28 p4510 samples), and samples used to select genes for training (an additional 4 healthy donor samples), internal independent validation set samples (5 healthy donor samples and 33 samples from 4 other CTCL patients), and external validation set samples (8 patient samples from the NIH).

### 2.11. Ingenuity Pathways and DAVID Functional Enrichment Analyses

Pathway and functional analysis were carried out with Ingenuity Pathways Analysis software (version 8.0, Qiagen, Redwood, CA, USA; http://www.ingenuity.com/ (accessed on 3 February 2022)) using Ingenuity Core Analysis. FDR levels were estimated according to the Benjamini–Hochberg procedure. Enrichments of gene ontology (GO) terms, KEGG, and BIOCARTA pathways, along with Swiss-Prot, INTERPRO, and SMART keywords in a gene list, were assessed using DAVID software (version 2018, DAVID Bioinformatic team, Frederick, MD, USA). Results were filtered to satisfy an FDR < 20% criterion.

### 2.12. Transcription Factor Binding Site Analysis

Enrichment of transcription factor binding sites was carried out with DAVID software [37] with default parameters. DAVID treats instances for genes to have binding near each gene separately, so there is a possibility of multiple genes sharing the same motif region. The nominal *p*-value was corrected for multiple testing using a Bonferroni correction.

### 2.13. Identification of miRNA Target Genes

Putative target genes for various miRNAs were identified using the Ingenuity Knowledgebase of experimentally confirmed miRNA targets and/or those computationally predicted with high confidence by TargetScan software (version 7.2, MIT, Cambridge, MA, USA) [38].

### 2.14. Statistical Tests

The following tests were used to determine the significance of results under a threshold of *p* < 0.05. Spearman correlation: Concordance of methylation and miRNA data with the proportion of malignant cells in a given sample at a given time was tested using Spearman correlation. Pearson correlation: Concordance of the predicted vs. observed percentages of malignant cells for the training and validation sets was determined by the Pearson correlation test. Fisher exact test: Significance of enrichments was estimated using the Fisher Exact test. Hypergeometric test: Significance of overlaps between two groups of genes (with A and B number of genes) selected from the same pool of C expressed genes was tested using the hypergeometric test. The expected number of overlapping genes was calculated as (A × B)/C.

## 3. Results

### 3.1. Study Population and Assays

The basic clinical characteristics and parameters for the five patients undergoing romidepsin treatment and four heathy donors used in the study are summarized in Figure 1A. The study population included subjects of different races and patients with CTCL of various stages and responses to treatment with the same subject visits reaching up to 30. The sample collection for patients (p4502, p4503, p4507, p4509, and p4510) was performed at HUP [32] and healthy donors at the Wistar Phlebotomy Facility. For patients with CTCL, the term “visits” refers to romidepsin intravenous infusions received on days 1, 8, and 15 of every 28-day treatment cycle for the number of treatment cycles a patient completed in the study. Blood was collected at each visit before initiation of the romidepsin treatment and after completion of treatment. Repeated samples from healthy donors were similarly collected at weekly intervals of 2 to 5 weeks to match the collection protocols. Collected samples were used to assess the abundance of relevant lymphocyte populations (Supplementary Figure S1A), and isolated peripheral blood mononuclear cells (PBMCs) were assayed using Illumina WG-6v2 human whole-genome arrays for mRNA gene expression, Illumina MicroRNA v2 for miRNA expression studies, and the Illumina 27 k human methylation array to assess DNA methylation (Figure 1A, Supplementary Table S1). Resulting data for each subject included a combination of up to four types of measurements: cell counts, gene expression, miRNA expression, and DNA methylation.

The focus of the study was initially placed on patient p4510, classified as a complete responder with sequential samples collected over 12 cycles of romidepsin treatment. Samples from selected treatment cycles were used to assess tumor burden based on the fraction of circulating CD4^+^CD26^−^ malignant cells measured by flow cytometry. When beginning treatment, p4510 exhibited whole-body erythroderma, with 93% of lymphocytes having a CD4^+^CD26^−^ phenotype and a CD4:CD8 ratio of 64:1. All blood-based indicators of disease stabilized at normal levels by cycle 7 of treatment with the reduction of the CD4–CD8 ratio from 64 to 1.1 and that of the CD4^+^CD26^−^ population to normal levels (<5% for CD4^+^ T cells/< 20% for lymphocytes) [32]. These indicators (including clearance of all skin symptoms) remained at normal levels from cycle 7 to the final treatments at cycle 12. Disease recurred 9 months after treatment cessation, and 1 month after, the relapse sample was taken. Empirical levels of circulating malignant cells as determined by flow cytometry (FACS) and/or microscopy were available for nine of the p4510 treatments that spanned the 12-cycle period and for the one sample taken after the recurrence.

### 3.2. Markers of Malignant Cells

We initially took advantage of the large number of samples from the complete responder patient p4510 to acquire insight into the effects of treatment on the malignant cells. This patient had (1) 12 cycles of treatment with PBMC samples from each cycle and FACS data from various intervals during the 12 treatment cycles. (2) The 12 cycles included a period of clinical remission, but with a persistence of residual disease supported by a later relapse. (3) The relapse sample was available for comparison with a sample from each stage of treatment. We first analyzed the nine samples where the numbers of circulating malignant cells had also been monitored by flow cytometry and microscopy (Figure 1B). The figure demonstrates a gradual reduction of the proportion of malignant cells with progressive romidepsin treatments. The data for the relapse sample is included to demonstrate the increase in the malignant cell count 10 months after treatment termination and 1 month after detection of the relapsed malignant cells.

**Figure 2:**
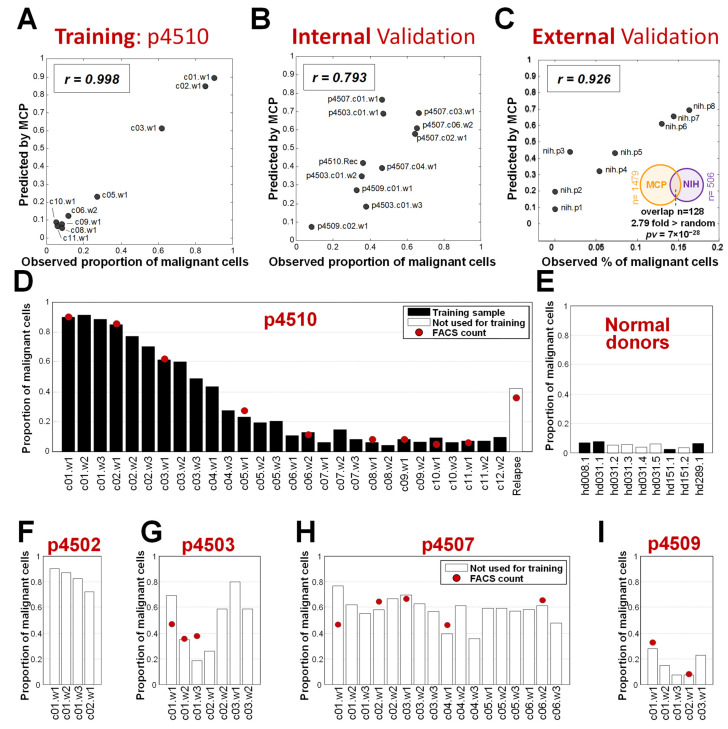
Proportion of malignant cells predicted by the MCP. (**A**) Correlation of observed and MCP performance on the p4510 training samples. (**B**) Application of MCP on an independent validation set of additional romidepsin-treated patients with observed cell counts. (**C**) Correlation of MCP performance with observed cell counts on an external validation set from the NIH study for MF patients (Supplementary Table S2) and significance of overlap between MCP genes and genes derived from the study. (**D**–**I**) Predicted MCP values for all time points for patients and healthy donors. Red dots indicate values measured by flow cytometry.

The consistent decrease in malignant cells allowed us to apply a Gaussian model (R^2^ = 0.998, Supplementary Methods, Supplementary Figure S1B) fit to the flow cytometry data from the nine visits and then to extrapolate the malignant cell proportions for all 28 treatment visits, including those where cell count data was not available. The resulting malignant cell proportions were used with p4510 mRNA expression data to define the panel of genes over-expressed in malignant cells. We first identified genes whose expression was significantly (FDR < 5%) positively correlated with the changing proportion of malignant cells associated with progressive romidepsin treatments. This resulted in a signature of 1479 malignancy-specific genes, predictive of the relative levels of malignant cells in a sample, referred to as the “malignant cell predictor” (MCP) (see Section 2 and Supplementary Methods for additional details). The nine samples from patient p4510 that were used to initially create the signature exhibited, as expected from a training set, a high correlation between the proportion of malignant cells predicted by MCP and the observed numbers determined by FACS (Pearson *r* = 0.998) (Figure 2A). When the MCP was applied to a set of 10 independent validation samples from the four additional patients with measured circulating tumor burdens, the MCP-predicted values also highly correlated with the observed malignant cell proportions (Pearson *r* = 0.793) (Figure 2B).

We also applied the MCP to gene expression in the RNA collected prior to the start of treatment from eight patient samples treated with romidepsin at the National Institutes of Health (NIH). All eight patients were diagnosed with skin mycosis fungoides (MF) and seven of the eight also had low-to-medium levels of CTCL blood involvement [39,40]. The circulating malignant cell numbers predicted by the MCP had a correlation of r = 0.926 with the numbers determined by FACS (Figure 2C), indicating accurate estimation of the number of circulating malignant T cells in the samples from MF patients. In addition, 25% of the genes significantly correlated with tumor burden in MF patients were present in the MCP gene list, a significant overlap 2.8 fold more than expected by chance (*p* = 7 × 10^−28^ by hypergeometric test, Figure 2C). A number of publications have focused on the differences between MF and circulating SZ cells, including that by Clarke et al. [41] and more recently, that by Miyagaki and Sugaya [42]. Our results suggest that MF cells that progress from skin-associated MF to leukemic CTCL do share some gene expression relationships with Sézary cells from patients with no apparent skin involvement. This may be because our analysis was cell-type blind and not focused on cell-type-specific markers, so we were able to capture this shared signature.

As the MCP signature demonstrated an accurate assessment of samples with low-to-high levels of circulating malignant cells from a variety of patients with different levels of circulating cells, we then used the MCP to assess changes in the levels of malignant cells in patient samples including those that lacked FACS data to approximate changes over successive romidepsin treatments. We also analyzed samples from healthy donors. We applied the MCP to all samples with mRNA gene expression data available in the romidepsin study, including 28 on-treatment samples from patient p4510 and a sample taken after recurrence, 4 healthy donor samples used in the MCP development, 33 samples from the four additional phase II patients, and 5 additional samples from healthy donors that were not used in the MCP model design. This analysis provided several important observations. First, compared with the FACS measurements for patient p4510 samples, the MCP showed accurate estimations (Figure 2D), including detection of malignant cells in the recurrence sample, indicating the robustness of the signature for the prediction of developing recurrence. Healthy donor samples (Figure 2E), as expected, had levels similar to clinically undetectable malignant cell counts. Application of the MCP also provided a more complete picture of the treatment responses over time for the four other patients. Patient p4502, clinically classified as a non-responder, had no associated FACS data but was predicted by the MCP to have 90% circulating malignant cells at baseline (Figure 2F). This patient withdrew from the study after the first cycle of treatment due to side effects. Based on our predicted assessment of circulating malignant cell levels, the cycle 1 treatments had a relatively large initial effect on the circulating malignant cell numbers, with a projected 18% reduction of malignant cells. Patients p4503, p4507, and p4509 (Figure 2G–I) were all classified as partial responders. While patients p4503 and p4509 initially exhibited significant positive effects from the treatment, as evidenced by a reduction in circulating tumor cell numbers, unlike the progressive reduction in malignant cells seen with p4510, this initial effect was reversed by the second and third treatment cycles, respectively, indicative of emerging treatment resistance. Both patients withdrew from the study during cycle 3, patient p4509 because of side effects and p4503 due to progressive disease. Patient p4507, with stable disease, remained on treatment for the full six cycles but exhibited little change in circulating tumor burden according to FACS or MCP assessment (Figure 2H).

In summary, the MCP established using 28 samples from patient p4510 and initial samples from four normal donors, was validated on an independent internal set of 1 relapse samples, 33 samples from four additional patients, 5 samples from healthy donors, and external samples from eight MF patients.

### 3.3. Functions and Processes Associated with Malignant Cells

**Figure 3:**
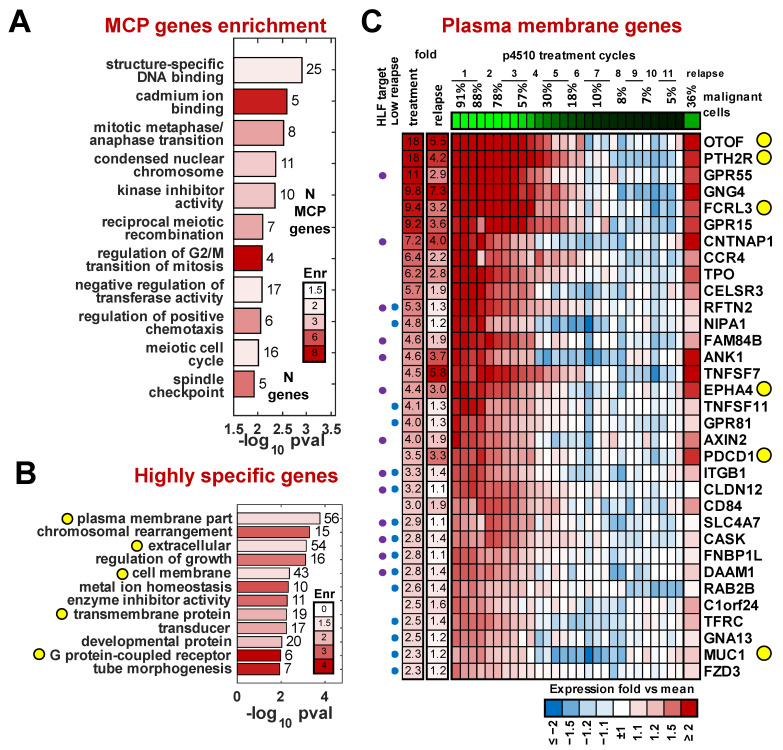
Gene categories dysregulated in the malignant cell. (**A**) Categories significantly enriched among 1479 MCP genes. (**B**) Categories significantly enriched among 291 MCP genes most specific to malignant cells (fold > 2). Bar numbers indicate numbers of significant genes in that category; yellow circles indicate potential surface markers categories. (**C**) Heatmap of expression for MCP genes annotated with plasma membrane and receptor activity GO categories. Purple/blue circles mark HLF target and low relapse genes respectively. Yellow circles indicate genes of interest discussed in the Results.

To identify biological processes potentially altered by the MCP expression profile, we analyzed the 1479 MCP genes using DAVID software [43]. We observed several enriched categories related to meiosis/mitosis, kinase inhibition, metal ion and DNA binding, and chemotaxis (Figure 3A and Supplementary Table S3). When performing enrichment within a more conservative subset of 291 of 1479 MCP genes that changed at least 2-fold during treatment, we found the plasma membrane category to be the most significantly overrepresented class (Figure 3B), (*p* = 2 × 10^−4^, FDR = 0.21%). This is a gene set of particular interest for potential use as a marker to identify, isolate, or target the malignant cells.

To identify potential markers that might be expressed on the cell surface, we searched for plasma membrane (GO:0005886) or receptor activity (GO:0004872) matches among all 1479 MCP genes. We further filtered this list by applying four additional criteria associated with their differential expression between the patients and healthy controls: (1) the magnitude of upregulation in malignant cells (based on data from patient p4510) of at least 2-fold, (2) significant upregulation in the pretreatment samples compared to the average expression in healthy donors for the four additional treated patients (*p* < 0.05), (3) significant expression difference (*p* < 0.05) between purified CD3^+^CD4^+^CD26^−^ and CD3^+^CD4^+^CD26^+^ cells from three independent Sézary syndrome patients of at least 1.2-fold (Supplementary Figure S2), and (4) a significant gene expression change (*p* < 0.05) in purified CD3^+^CD4^+^ cells from three TCR VB monoclonal Sézary syndrome patients compared to CD3^+^CD4^+^ cells from three healthy donors described in Wysocka et al. [33]. This resulted in a list of 33 genes associated with malignant cells that were annotated to have plasma membrane or receptor activity function. We also assessed the level of the re-expression of these genes in the recurrence sample compared to expression levels at various times during treatment (Figure 3C).

Several of the top candidates included genes normally not expressed in lymphoid cells. The most differentially expressed of these genes was the deafness-associated gene otoferlin (OTOF), with highly restricted expression in the ear [44]. Additional non-lymphoid genes included parathyroid receptor 2 (PTHR2) and thyroid peroxidase (TPO), both of which have been connected to autoimmune disorders. PTHR2 expression has also been associated with hematopoietic stem cells [45]. We recently identified FCRL3, an orphan receptor expressed by lymphocytes and implicated in several autoimmune diseases, as a marker for the presence of the Sézary T cells in patients with high and medium tumor burden [33]. Programmed cell death 1 gene, PDCD1 (PD-1) [46], previously shown to be expressed in Sézary cells, is included in this list. It was also detected in the recurrence. PD-1 regulates cell proliferation and transformation by modulating the ERK pathway [47]. Notably, PD-1 expression in the recurrence sample reached similar or slightly higher (9%) levels of PD-1 in the pre-therapy sample with only 1/3 of the number of malignant cells, suggesting expression levels on a per-cell basis may be increased. Although evidence from PD-1-targeted immunotherapy in CTCL is limited, a recent study suggested its potential utility [48]. Other genes in this panel that follow this same behavior included CELSR3, a non-classic member of the cadherin superfamily that does not bind catenin, and MUC1, a sialo-glycoprotein frequently expressed on solid tumors [49]. Targeting the Muc1 c-terminus has been shown by Jain et al. [50,51] to induce ROS-mediated death in CTCL. Of note is the observation that a number of genes highly expressed in the pretreatment and early treatment samples were not significantly re-expressed in the recurrence sample. There were several plasma-membrane-associated genes that did not show high re-expression (<1.5-fold) in the relapse sample and are likely not associated with resistance but may be associated with response to romidepsin (Figure 3C).

### 3.4. Mechanisms of Dysregulation of the MCP Genes

We then sought to determine which mechanisms might be responsible for the dysregulated gene expression in the malignant cells. To address this, we analyzed three major levels of transcriptional regulation: (1) the presence of transcription factor target sequences, (2) DNA methylation patterns, and (3) miRNA expression profiles. We assessed the complete list of 1479 MCP genes and the more robust subset of 291 MCP genes with a change in expression of 2-fold or more. While several studies have examined epigenetic changes in CTCL including miRNA expression and DNA methylation, as reviewed in [52], we specifically focused on patterns associated with our MCP signature and how they change in response to treatment with romidepsin.

**Figure 4:**
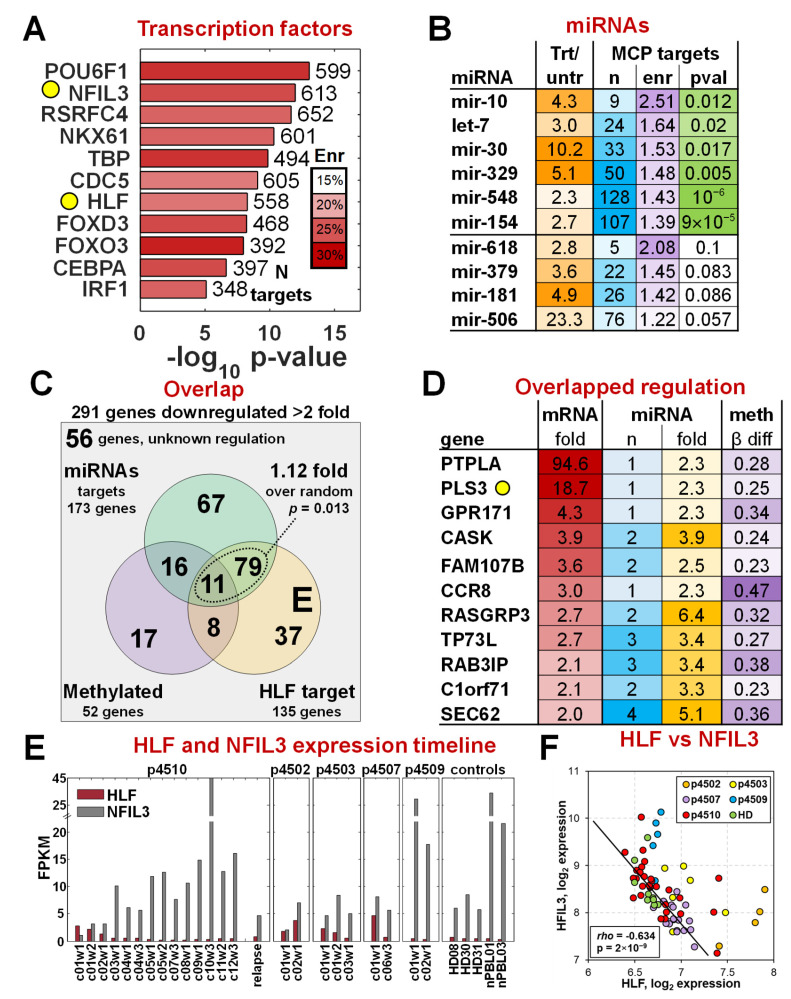
Regulation of malignant cell gene expression. (**A**) Transcription factors (TF) with binding sites significantly enriched among the 1479 MCP gene set. HLF was the only TF that also ha mRNA expression changes that support its activity in the malignant cells, and NFIL3 has the same motif binding site as HLF, as highlighted in the yellow circles. (**B**) List of miRNAs with changes in expression levels that correlate with the percentage of malignant cells present in a sample. The top 6 miRNAs that have their targets significantly enriched in the 291 MCP genes with >2-fold changes. (**C**) Overlap between HLF targets, miRNA targets, and changes in gene methylation patterns. (**D**) Eleven genes with HLF target sequences, target sequences for at least one of six top miRNAs, and DNA methylation differences altered during treatment. PLS3, found in previous studies to be a marker of CTCL in a subset of patients, is highlighted with the yellow circle. (**E**) mRNA expression changes of HLF and NFIL3 mRNAs as a function of romidepsin treatments. (**F**) Spearman correlation between HLF and NFIL3 expression levels.

Using DAVID enrichment analysis software [43,53], we identified 11 transcription factors that had significant enrichment of their binding motifs near the 1479 MCP genes (Figure 4A). Among these factors, only HLF (hepatic leukemia factor) had associated expression changes at the mRNA level in addition to a significant enrichment of at least 20% (Bonferroni-corrected *p*-value = 10^−6^) of its binding motif sequence present in those genes, with the significance and enrichment remaining stable up to a 10 kb distance (Supplementary Figure S3). Among the 11 factors, we found NFIL3 (nuclear factor, interleukin-3 regulated), also a member of the PAR bZIP family of transcription [54]. Both HLF and NFIL3 share the same DNA binding motif and are involved in regulation of circadian rhythms, cell death, and drug metabolism, with HLF acting as a transcriptional enhancer (oncogene) and NFIL3 as a transcriptional repressor (and sometimes tumor suppressor). We identified 577 of the 1479 MCP to have an HLF/NFIL3 binding motif in the vicinity (Figure 4A) and found that 135 of these genes were downregulated 2-fold or more, with progressive romidepsin treatment suggesting that HLF plays a potential major role in their upregulation in the malignant cells.

miRNA expression (723 miRNAs and 355 unique families) was analyzed on the Illumina MicroRNA v2 Sentrix Beadchip using 16 samples from patient p4510 and identified 24 miRNA families with significantly increased expression of at least 2-fold (FDR < 0.1%) during treatment (Figure 4B), suggestive of an association with the reduction in malignant-specific gene expression. All had mRNA targets that were either experimentally confirmed or predicted with high confidence by TargetScan [38]. miRNA targets for six of the miRNA families were identified in 260 of the 291 highly expressed MCP genes (Figure 4B, Supplementary Table S4), a number significantly higher than random (*p*-value = 0.025) as determined by the hypergeometric test. Overall, there were 173 genes predicted to be targeted by at least one of the six miRNAs, with 52 of the genes being regulated by three or more of the six (Supplementary Figure S4). Five genes, ZNFN1A2, ST8SIA1, PGM2L1, PHC3, and TMEM2, were predicted to be regulated by at least five of the six miRNAs.

We also assessed the methylation status of the 291 MCP genes on Illumina 27 k methylation arrays for the 17 treatment time points of p4510. Methylation was previously shown to play a part in dysregulation of several CTCL-associated genes including PLS3 [8,55,56]. We found that 52 of the 238 MCP genes (Supplementary Figure S5) that had methylation data on the 27 k array platform had a significant (at least 20% change, FDR < 5%) decrease in methylation in the malignant cells (observed changes during treatment from 0.23 to 0.61), a number higher than expected by chance alone (*p*-value = 0.0096 by hypergeometric test). The combined analysis of transcription factor binding sites, miRNA expression, and DNA methylation changes revealed that higher HLF, downregulation of six of the miRNAs, and de-methylation of a subset of genes had a non-random impact on the increased mRNA expression of MCP genes in the malignant cells. The overlap of effects between the three regulatory mechanisms for the 291 MCP genes is shown in Figure 4C. Although the methylation effects did not quite reach the significance threshold (*p* = 0.136, *p* = 0.082 respectively) with either miRNA or HLF regulation, all genes regulated by any of the top six miRNAs also had a HLF binding site (1.12 higher, *p* = 0.013). It should be noted that four of the six miRNA families (let-7, mir-154, mir-30, mir-548) are also predicted to regulate HLF with high confidence by TargetScan. Thus, a subset of the MCP genes could be downregulated in normal cells by direct targeting by those miRNAs and a secondary effect of the miRNAs downregulating the HLF transcription factor that is driving their transcription. Eleven genes showed evidence of regulation by all three mechanisms (Figure 4D), including PLS3 and several other genes not normally expressed in PBMCs.

The analyses identified HLF as a key positive regulator of a significant number of malignancy-specific MCP genes. HLF and NFIL3 have been shown to have important regulatory functions in a number of solid tumors as reviewed in Ahmadi et al. [57] with a role in promoting ferroptosis resistance in triple-negative breast cancer [58]. Except for HCC, HLF is downregulated in all solid tumors [57]. While HLF is a transcriptional enhancer, NFIL3 has been shown to repress gene expression of their shared targets by recruiting histone deacetylase 2 and G9a histone methyltransferase [59]. NFIL3 is highly expressed in normal Th2 T cells and is required for TH2 T-cell functionality [60]. Figure 4E shows the relative expression levels of HLF and NFIL3 as a function of successive romidepsin treatments, and a statistical summary is shown in Figure 4F. Four of the five patients also had levels of HLF expression that were significantly higher than those in healthy controls. HLF levels dropped significantly as treatment advanced and the malignant cell number dropped. While NFIL3 levels were barely detectable at the start of p4510’s treatment, they increased to very high levels at the end of the cycle 10 treatments. These levels decreased again by 80% in remission cycles 11 and 12 as HLF expression again increased, suggesting increasing residual disease. The NFIL3 expression was even further decreased in the recurrence sample with a proportional increase in HLF. NFIL3 expression was more variable among the other four patients, further emphasizing patient heterogeneity and their differences in response to romidepsin treatments. Partial responders p4503 and p4507 also showed a reduction of HLF levels and an increase in the expression of NFIL3, while stage IVA non-responder p4502 showed only a modest increase in NFIL3 although HLF increased significantly. Partial responder p4509 is clearly an outlier, with an expression of NFIL3 and HLF more similar to that of controls, although both HLF and NFIL3 transcripts did decrease slightly with treatment. The overall highly significant negative correlation of transcriptional enhancer HLF and transcriptional repressor NFIL3 across samples with different proportions of malignant cells (Figure 4F) demonstrates opposite “yin-yang” roles of HLF and NFIL3 in the malignant cells. Although this study is relatively small, the robust HLF-to-NFIL3 ratio across the CTCL patients and donors is compelling evidence supporting its potential utility in monitoring romidepsin treatment effects and more general application in detecting malignant cells and might be a target for further focused studies.

### 3.5. A Gene Expression Signature of Residual Disease

**Figure 5:**
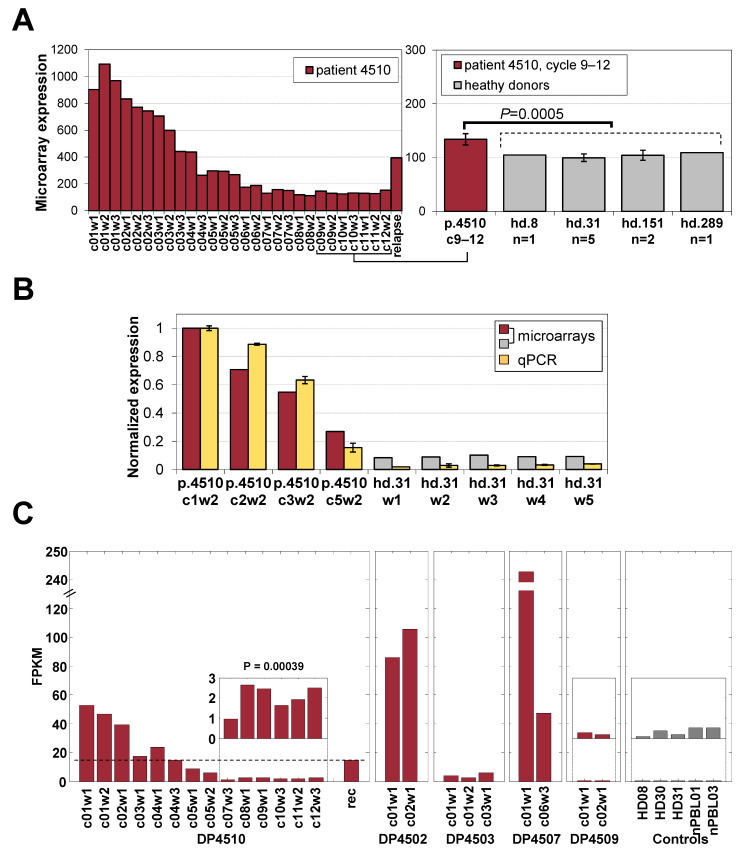
Expression of PLS3 in healthy donors and patients. (**A**) Expression of the malignant-cell-specific PLS3 gene in samples from p4510’s treatment and after recurrence, including a direct comparison of cycles 9–12 samples to those of 4 healthy donors from microarrays. (**B**) PLS3 expression from microarray compared to that by qRT-PCR. (**C**) RNA-seq data for PLS3 expression in all 5 patients showing contrasting with the PLS3-negative status in p4509 and healthy donors.

To assess the ability to detect the presence of small numbers of circulating malignant T cells during remission in patient p4510, we focused first on the expression of PLS3 which was not expressed in normal blood cells (Supplementary Figure S6) and was shown above to be highly specific to the samples with a high percentage of malignant T cells (18.7-fold change during treatment, Figure 4D). PLS3 had significant changes in methylation values from an average of 9% methylation to an estimated value of 24% at later cycles 6–12, when the patient was considered free of disease. This suggests that hypo-methylation may be an important factor in the expression of this unique marker, as previously shown [8]. We found small but statistically significantly higher PLS3 expression levels (28% higher, *p* = 0.0005 by *t*-test) in the remission samples compared to healthy donor controls (Figure 5A). We validated this difference using qRT-PCR (Figure 5B) and with RNA-seq on selected p4510 samples across the period of remission and recurrence (Figure 5C), confirming PLS3 levels across remission treatment cycles (c7–12) to be significantly higher (*p* = 0.0004) than the levels detected in healthy donor controls and demonstrating the PLS3 levels in the relapse/recurrence sample matched treatment cycle 4 during week 3, when the patient had similar numbers of malignant cells (Figure 5C). However, this correlation of PLS3 levels and malignant cell numbers was not as evident in all the other patients, with patient p4503 having levels only slightly higher than those of the p4510 remission samples and p4509 having levels similar to those of healthy donors. Patients p4502 and p4507 had PLS3 expression levels higher than those of p4510, and although these levels decreased by cycle 6 of treatment, the levels only dropped to the pretreatment or early treatment levels detected in p4510. Although single markers such as PLS3 may be informative for patient subsets, we had hoped to find a set of markers common to most patients that might be representative of early events in malignant cell development and not detected in the healthy donors.

Patient p4510 was clinically assessed as having a complete response by the end of treatment cycle 6, the designated endpoint for the trial, with a resolution of skin disease, no evidence of abnormal lymph nodes, and normal levels of circulating CD4^+^CD26^−^ cells (~5–10% of CD3^+^CD4^+^ cells and a normal CD4:CD8 ratio) [32]. The patient elected to stay on treatment for an additional 6 months, providing the opportunity for continued monitoring during the remission period, including treatment cycles 7–12 when treatment was stopped for an unrelated condition. The patient was diagnosed with a recurrence 9 months after stopping therapy. We then sought to determine whether evidence for residual malignant cells could be detected in the gene expression data for the elected treatment cycles 7–12. Based on MCP prediction, we detected a few significant changes in cycles 9–12 in the last three treatment cycles (Figure 2D). We compared the gene expression for those time points to the expression levels in the four healthy donor samples. This approach assumed that if residual malignant cells were present, they would retain the deregulated expression of at least a subset of the original 1479-probe MCP signature and if sufficiently unique to the malignant cell, could be detected by differential expression analysis even if very few cells remained.

**Figure 6:**
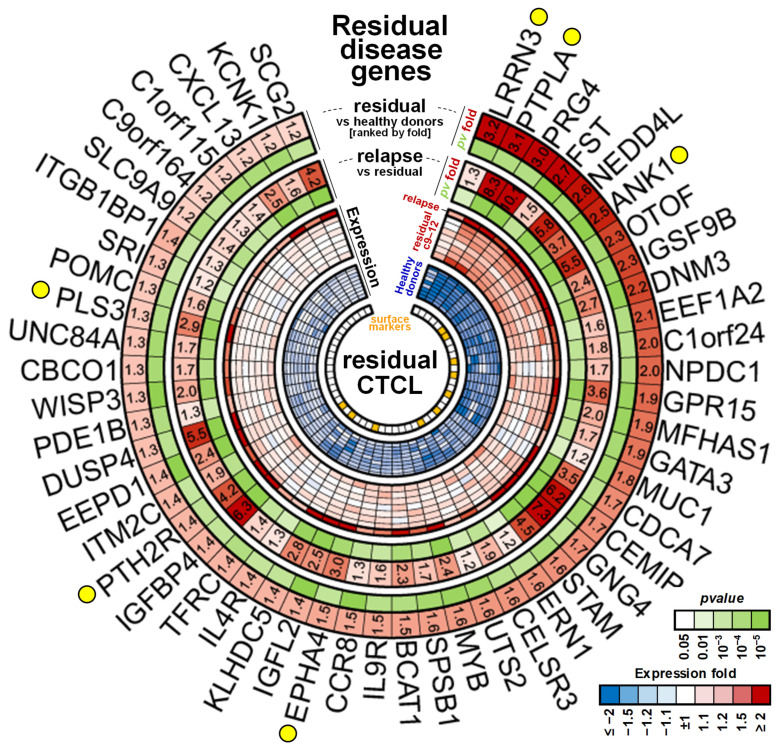
Markers associated with residual disease. Expression heatmap of the 52 genes detected at significantly higher levels in remission samples as compared to the normal levels of healthy donors (at least 1.2-fold) and that showed an increase in the relapse sample 9 months after therapy (at least 1.2-fold). Yellow circles indicate genes of interest discussed in Section 3. Orange squares in the inner circle show candidates overlapping with plasma membrane proteins from Figure 3C.

Of the 1479, we identified 52 unique MCP genes (Figure 6, Supplementary Figure S7) with expression levels that were still significantly higher (at least 1.2-fold and FDR < 5%) in the remission samples compared to the healthy donor controls and also showed an additional significant (FDR < 5%) increase in expression of at least 20% after cancer relapse, with 39/52 of these showing a >1.5-fold increase indicative of residual disease. A number of the residual disease markers had been previously associated with CTCL cells including four highly diagnostic genes we and others described previously as distinguishing the normal and malignant cells, including Gata3, PLS3, and DUSP4 [6,7]; the microtubule-associated GTPase DNM3 [61], the c-MYB protooncogene [62]; and the ephrin receptor EPHA4 [63]. Of additional interest are a number of newly identified and highly differentially expressed genes in this signature that like PLS3 are not normally expressed in lymphoid cells and/or are only expressed early in development. These include the following: (1) the highly conserved leucine-rich repeat neuronal protein 3 (LRRN3), normally highly expressed in fetal brain [64]; (2) proteoglycan 4 (PRG4), normally expressed in chondrocytes [65], with the F Isoform acting as a growth factor for primitive hematopoietic and endothelial cell lineages [66]; (3) the heart-specific protein phosphatase PTPLA [67], also found to be highly malignancy-specific (see Figure 3B); (4) the parathyroid-hormone-specific receptor PTH2R expressed in skin [68] and in hematopoietic and AML stem cells [69]; and (5) otoferlin (OTOF), highly specifically expressed in the inner ear and associated with auditory defects [70].

### 3.6. Detection of Residual Disease

The detection of a residual disease signature that persisted through the additional six cycles of treatment indicated that sub-population(s) of CD4^+^CD26^−^ malignant cells with different sensitivities to HDAC inhibitor treatment were present in patient p4510, and as indicated by MCP validation, in other Sézary patients. To address this possibility, we carried out flow cytometry on a subset of the markers we identified that were part of the residual disease signature and were expected to be detectible on the cell surface. We focused on membrane-associated proteins that were also expressed in the recurrence sample with available antibodies that could be used for flow cytometry. We also included CD164, a sialomucin (MUC-24) that is also a member of the MUC family, that we recently showed to be differentially expressed on the surface of malignant CD4^+^CD26^−^ Sézary cells [33] and was also identified as part of the 1479 MCP gene list identified in this study. Although CD164 is widely expressed in lymphocytes, it is only rarely expressed on the cell surface although surface expression has been associated with some activated T cells and hematopoietic stem cells [71], suggesting that the mRNA expression and protein expression by FACs could differ. We also assayed for PD-1 expression as it has also been associated with the CTCL T cell [46] and is a potential target for therapy. Additional residual disease markers for which flow tested antibodies were available were MUC1, reported to be expressed by hematopoietic stem cells [72,73,74]. Since we had access to only limited numbers of viable frozen cells from our five patients, we were limited in the numbers of markers we could test. We also analyzed viably frozen samples, some taken prior to or after romidepsin treatments, where available, as well as cells from a selection of 26 independent patients with low-to-high circulating tumor burdens, and 10 gender- and age-matched healthy controls. The flow cytometry results are summarized in Supplementary Figure S8. The percentage of lymphocytes that were CD4^+^CD26^−^ in the healthy donors ranged from 5 to 15% and that of CD4^+^CD164+ ranged from 11 to 40%. Surprisingly, we found CD164 was more frequently expressed on the surface of CD26^+^ cells than on the CD26- cells of healthy donors. This same pattern was evident for the low-tumor-burden (LTB) patients, those with <20% CD4^+^CD26^−^ T cells as defined by Benoit [75]. CD164 expression on CD26+ cells was on average, 3 fold more than its expression in CD4^+^CD26^−^ cells in 12/12 of the LTB patients tested. This trend was repeated in 3/6 of the medium-tumor-burden (>20% MTB) patients with the other 3 being similar to those with a high tumor burden (HTB) (>50%). In HTB patients, the CD26^−^CD164^+^ cells were, on average, 10 times as common as the cells with CD4^+^CD26^+^CD164^+^. We found that CD164 was uniformly expressed at the mRNA level on CD26^−^ and CD26^+^ cells, but the protein was only detectable on the surface of specific subsets of cells, with the percentages of CD26^−^CD164^+^ cells increasing with increasing tumor burden. Based on RNA-seq analysis, the mRNAs proteins were normal, and mutations do not account for the different patterns of localization in LTB vs. HTB patients.

Initially we detected very little surface expression of PTHR2 or MUC1 protein on the malignant cells. The PTHR2 results were similar to those of MUC1, but as the signal was quite low, we abandoned this marker and instead re-analyzed a subset of permeabilized patient and control cells for the combined presence of intracellular MUC1 and CD164 (Supplementary Figure S8). While CD164 was detected across all CD4^+^ cells after permeabilization, consistent with the gene expression results, MUC1 was found to be primarily detectible only in the CD4^+^CD26^−^ T cells as previously shown by Jain et al. [50,51]. We expect that the PTHR2 protein will behave similarly to MUC1.

## 4. Discussion

While most genomic studies of drug response are carried out in vitro, we have followed the process of response in vivo by sampling the peripheral blood of five patients with CTCL being treated with the HDACi romidepsin at HUP. Samples were collected before and after each successive treatment. The proximity of the laboratory to the treatment center enabled us to collect and process samples in a timely manner critical to obtaining results true to what is happening in vivo. In addition, the samples from healthy donors were also collected at the same time of day as that of patient sample collection. Our intent in this analysis was not to just find a few genes that distinguish malignant from non-malignant cells but to develop a more complex overview of the transcriptional programs that define the origins of the malignant cell and begin to understand the complex molecular changes related to romidepsin treatment. We followed gene expression changes in five patients that received from 4 to 38 successive romidepsin treatments. The center of our study is patient p4510 classified as a CR as defined by Kim et al. [32]. Patient p4510 was followed through for 12 months of treatment, including a 6-month period of remission, and an eventual recurrence arose 9 months after treatment ended. This patient was the model/prototype for the development of a malignant cell predictor (MCP), a panel of 1479 genes that allowed us to correlate gene expression data with changing circulating tumor burdens in response to romidepsin treatment. The MCP includes many genes previously reported to be over-expressed in comparisons of gene expression profiles of PBMCs from patients with CTCL vs. those from healthy donors (e.g., GATA3, PLS3, TNSFS10, DUSP4, and MYB) as well as newly identified dysregulated genes whose expression is normally excluded from lymphoid cells suggesting extensive epigenetic changes in the malignant T cell. Although patient heterogeneity is significant, this panel was able to capture a signature shared by patients from different locations and with significantly different tumor burdens and clinical histories. A subset of the MCP genes can detect the presence of small numbers of residual malignant cells or early-stage evidence of a recurrence in apparently disease-free patients, an important goal in patient management. While limited in the number of patients under consideration, our analysis approach allowed us to validate the MCP on an independent set of 47 samples that were not used in its development and included relapse, 5 samples from healthy donors taken at different times, samples from four patients from an internal study, and samples taken from 8 MF patients.

Single-cell studies of in vitro treated cells described in Buus et al. [76] demonstrated that “circulating malignant cells display a high degree of heterogeneity within the malignant T-cell population and include discreet subpopulations of T cells that carry HDACi resistance”. This diversity is also evident at a global level in this and our previous studies. While the responses to romidepsin varied for the four other patients being treated in this trial (Figure 2D–I), they all showed evidence of the presence of both drug-sensitive and drug-resistant malignant subclones. For example, high-tumor-burden patient p4503 had a 50% reduction in circulating malignant cells after one treatment and a 75% reduction after two treatments. Unlike the progressive response and remission seen with p4510, this highly significant response was short-lived, with malignant cell levels returning to pretreatment levels by the fifth treatment. Patient p4503 was one of the few patients in our study with almost 100% of the CD4^+^CD26^−^ cells being positive for both cd164 and PD-1. Patient p4509 (MTB) had a similar rapid response and recurrence by treatment 9, while p4502 had a slow but consistent response similar to that of patient p4510 but unfortunately withdrew from the study after only four treatments. Patient p4507 (HTB) remained in treatment for six full cycles with stable disease and little change in malignant cell levels. In cases where the majority of the malignant cells were romidepsin sensitive, leading to apparent remission, continued treatment appeared to also be able to keep minor subclones in check if not eliminated, likely due to the recovery of some protective immune functions. Because of this heterogeneity of patients, we tried to identify a gene signature that is common to different variants and that may be representative of the primary changes leading to malignancy that are retained to some extent in evolving, diverse malignant subclones. The MCP signature must have captured some of that commonality as it accurately predicted malignant cell numbers compared to actual malignant cell counts in a variety of patient samples from different sources and different disease courses.

The residual disease signature, derived from the MCP, includes six genes associated with thyroid function and autoimmune syndromes not previously identified, including PTH2R, PTHLH, and genes related to calcium regulation in the body, and while not directly linked to CTCL, they have relatively high expression in the malignant cells and very-low-to-undetectable levels of expression in the normal control PBMCs. A potential connection may be through their influence on cell proliferation and differentiation, particularly in the skin where CTCL manifests. Otoferlin, a multirole Ca^2+^ signaling protein normally associated with deafness, is also unique to the malignant cells and one of the most highly expressed of the residual disease and recurrence signature genes. Recent studies have detected expression in B cell and plasma cells in diseases associated with muscle weaknesses [77]. Their lack of expression in the normal PBMC population suggests that they may be robust markers for the residual malignant cells. PGP4 [78] and tyrosine kinase receptor EphA4 [79] have been previously shown to be expressed in T cells of patients with CTCL.

In a previous study using FACS analysis, we found that CD164 expression on a large number of patient and control samples appeared to be specific to the CD4^+^CD26^−^ malignant cells [33]. This was of interest as CD164 could provide a positive marker for the CD4^+^CD26^−^ malignant cells and has also been associated with hematopoietic stem cells (HSCs). However, in our present study, using unfractionated PBMC RNA, we found that mRNA expression levels of CD164 in patient and control samples were quite similar as indicated by microarray and RNA-seq. The discrepancies between the mRNA and FACS suggest a specific population of patient cells can regulate the surface expression of CD164 [71]. The flow cytometry data performed in this study confirms the observation that surface CD164 is differentially expressed on lymphocytes from Sézary patients and that while surface expression is correlated with the presence of CD4^+^CD26^−^ cells in patients as well as in the small numbers of these cells found in healthy controls, it is also found on a subset of CD26+ cells. Using total PBMCs rather than purified CD3^+^ cells with the additional purification steps may account for some of the differences between the studies. The complexity of the evaluation of malignant populations by flow cytometry is described in detail in a recent article by Horna et al. [80], suggesting there might be significant overlap between reactive CD4 T-cells and Sézary cells in terms of CD164, CCR4, and CD279 expression. Differential surface expression of CD164 has also been linked to the expression of specific CD164 isoforms [71] not detected in our RNA-seq studies.

A recent review by Dulmage and Geskin [81] outlines the problems associated with various studies that focus on different populations of cells prepared by different methods. Our approach in this study was to first minimize the time from sample collection and PBMC isolation and to follow changes in gene expression as a function of treatment response as measured by a reduction in the malignant cell population. This provided the ability to define small numbers of lingering malignant cells after “clinically successful” treatment. We tried to deal with the issues of patient heterogeneity by attempting to find a class of dysregulated genes and processes that are informative within this heterogeneity, and we have been successful to some extent based on its applicability to a variety of different patients. The identification of potential new markers may provide new therapeutic targets and lead to the development of new reagents. Although this signature is based on the response to one specific treatment in one patient, it accurately detected the malignant populations in pretreatment samples from a variety of different patients with different levels of circulating malignant cells, suggesting it captures some level of commonality.

The analysis of potential interactions using data from different types of regulatory systems has provided new information on those interactions within the context of treatment. We identified, for the first time, an important role for the PAR bZIP transcription factor family members, HLF and NFIL3, primarily studied for their associations with circadian rhythms and solid cancers. While NFIL3 expression is normally relatively high in normal PBMCs, HLF is normally quite low. This ratio dominance of NFIL3 expression is diminished in the malignant cells with the increase in HLF expression. Both transcription factor proteins can act as homodimers or can heterodimerize and bind to the same DNA target but with different effects. HLF is a critical regulator of hematopoietic stem cell quiescence and circadian rhythms [57,82] and acts as a transcriptional enhancer and potential oncogene, while NFIL3 acts as a transcriptional repressor and potential tumor suppressor. It has also been shown to regulate expression of TH2 cytokines, IL-12, IL-4, and IL-15 [60], also associated with CTCL [83]. HLF expression has been previously noted in a study of gene expression in samples from MF patients [84]. We found that the increased expression of NFIL3 transcription, with the concomitant suppression of HLF transcription, in response to romidepsin, was highly correlated with the reduced expression of a large number of the MCP transcripts. Although there is little information on hematopoietic cancers, a recent pan-cancer study found that NFIL3 expression in cancerous tissues exhibited diminished levels when compared to normal tissue samples [85]. Although PBMCs from healthy donors vary significantly in levels of NFIL3 expression, in all cases, these levels were from 5 to 50 times the expression of barely detectable HLF in these samples, in contrast to what we found in our CTCL patient samples. Patient p4510 had the lowest detectable levels of NFIL3 pretreatment. suggesting target genes are dominated by HLF expression. NFIL3 expression increased almost 60-fold by remission treatment cycle 10 when HLF was barely detectable (Figure 4E). The levels of HLF also decreased significantly in response to treatment in patients p4503 and p4507, but the expression level of NFIL3 never significantly increased over the pretreatment levels. While patient p4502 had a similarly small increase in NFIL3 levels, there was no detectable decrease in HLF mRNA during the short time this patient remained on treatment, suggesting multiple regulatory pathways that regulate these genes were affected. Unlike the other patients, patient p4509 (MTB) had NFIL3 and HLF levels similar to those of healthy donors with higher levels of NFIL3. This patient also had PLS3 levels similar to those of controls. Despite these differences, the MCP accurately detected the malignant cell levels as measured by flow cytometry in all of the patients assayed.

NFIL3 is important in the regulation of genes involved in immune and inflammatory responses, among other processes. It functions as a homodimer but can also form heterodimers with parZip members HLF, DBP, and TEF, as well as CCAAT enhancer-binding proteins (CEBPs). CEBPα is one of the transcription factors with numerous binding sites in the MCP gene list. A recent study in neuronal cells [86] has shown that both NFIL3 and CEBPα/β share a preference for the TTACGTAA sequence. They also showed that NFIL3-bound genes are enriched in CEBP binding site, and that NFIL3 and CEBP TFs jointly regulate their gene expression with NFIL3, primarily acting as a transcriptional repressor. Additional associations have been found with FOXO [87], which is also in the list of transcription factors in Figure 4A. These observations suggest that NFIL3 functions are also moderated by other transcription factors that can be expressed at different levels. We focused on HLF because it was the only factor with a significant increase in expression in the malignant cells.

Multiple mechanisms interact to control gene expression in CTCL cells, as demonstrated by its significant associations with DNA methylation changes, miRNA abundance, and HLF binding (Figure 4). The mechanisms seem to be closely correlated, as we identified overlaps between different regulations of malignancy-specific genes (Figure 4C) that were more than expected by chance. Although DNA methylation effects did not quite reach significance in the overlap with miRNA or HLF regulation, the strongest overlap was observed between genes that had an HLF binding site and were targeted by any of the six reported miRNAs. Notably, the subset of four miRNAs (let-7, mir-154, mir-30, mir-548) are predicted to also regulate the HLF message levels. These findings paired with the identification of a significant number of miRNA targets among the MCP genes including HLF suggest interconnected effects of HLF activation and inhibition of miRNAs that result in the increased expression of CTCL-specific genes in the malignant cells. NFIL3 regulates diverse biological processes from circadian rhythm to cellular viability, as well as in immunological signal transduction, cancer, aging, and metabolism [87]. The effects of this competition between the NFIL3 repressor and the HLF activator for the same binding site is evident from the negative correlation of their relative expression levels and their target gene expression pattern changes detected from malignant cells (Figure 4F). These observations show that HLF and NFIL3 play important roles as transcriptional opposites in immune cell modulation and that their regulation may be critical to developing new or combination treatments for CTCL.

## 5. Conclusions

In summary, we identified a malignant cell predictor that captures a basic signature of the circulating malignant cells that accurately predicts levels of malignancy in samples from patients from a variety of sources and with significant differences in cancer stage, circulating tumor burden, previous treatment, and response to treatment with romidepsin. A subset of the MCP genes expressed in patient PBMCs have demonstrated potential for utility in the timely monitoring of residual disease and progression to recurrence. We also identify new transcriptional markers not normally expressed in PBMCs, such as otoferlin, that need to be further assessed. Importantly, we identified two transcription factors from the PAP bZIP family, HLF and NFIL3, that bind to the same DNA sequence with opposite effects, with target sequences in 500 of the MCP genes. Suppression of the expression of HLF and a significant increase in expression of NFIL3 were indicative of successful romidepsin treatment in this study. NFIL3 is a multifaceted transcription factor with a significant role in influencing the development and function of various important immune cell types. However, it is also a key factor in the development of autoimmune disease, and allergic reactions. A better understanding of the regulation and expression of these two transcription factors and how to balance their extensive opposing effects in the malignant cells may be an important new area for understanding, monitoring, and controlling CTCL.

## Data Availability

The data was submitted to the NCBI GEO database and is available under accession number GSE297556.

## Supplementary Materials

The following supporting information can be downloaded at: https://www.mdpi.com/article/10.3390/cancers17142380/s1, Figure S1: Cell populations and malignant cell fraction; Figure S2: Differences between CD3^+^CD4^+^CD26^−^ and CD3^+^CD4^+^CD26^+^ cells derived from Sezary Syndrome patients; Figure S3: Enrichment and its significance of HLF binding sites near MCP genes across varying distance parameter; Figure S4: Numbers of genes targeted by various numbers of miRNAs; Figure S5: Methylation changes among top changed MCP genes; Figure S6: Expression of PLS3 mRNA across multiple tissue types; Figure S7: Full list of 52 markers associated with residual disease; Figure S8: Flow cytometry measurements performed across different subjects; Table S1: Types of data that were used in the study for every patient; Table S2: Percent of blood involvement for patients, whose PBMC samples were obtained from NIH for the purpose of independent external validation; Table S3: Enriched categories in the list of 1479 MCP genes; Table S4: miRNAs that were significantly anti-correlated with malignant cell counts and show enrichment of predicted targets in the list of malignant-specific genes
