# Supplementary material for: Cutaneous T-Cell Lymphoma: Yin-Yang Effects of Transcription Factors HLF and NFIL3 in Regulation of Malignant T-Cell Markers in the Context of HDAC Inhibitor Romidepsin Treatment
Source: Cancers (Basel). 2025 Jul 17;17(14):2380. doi: 10.3390/cancers17142380 (PMC12293868; doi:10.3390/cancers17142380)
Supplement: Supplementary file 1 [file cancers-17-02380-s001.zip › cancers-3681137-supplementary.pdf]

Supplementary Methods

Calculation of the proportion of malignant cells in a CPT sample. Calculation of the proportion of malignant cells were done for samples that had (1) numbers of Monocytes (M) and Lymphocytes (L) counted manually by microscope and (2) numbers of T-cells (T, CD3+ marker), B-cells (B, CD3- CD20/22+ marker), NK-cells (NK, CD5-CD56/16+ marker) and malignant cells (Tm, CD4+CD7-CD26- marker) determined by flow cytometry. Proportions of malignant cells (PMC) was calculated as a product of the proportion of lymphocytes in a CPT sample (L/(L+M)) times the proportion of malignant cells among lymphocytes (Tm /(T+B+NK)):

$$PMC = \frac{\text{L}}{\text{L} + \text{M}} \cdot \frac{\text{Tm}}{\text{T} + \text{B} + \text{NK}}$$

PMCs were calculated for 9 time points for patient p4510 treatment and recurrent samples and 10 samples for 4 other patients. The *PMC*'s for the 9 time points of patient p4510 were used to fit a double Gaussian curve ($R^2 = 0.998$, **Supplementary Figure S1B**) as a function of weeks of treatment (*x*):

$$PMC = a_1 \cdot e^{-\left(\frac{x-b_1}{c_1}\right)^2} + a_2 \cdot e^{-\left(\frac{x-b_2}{c_2}\right)^2}$$

and extrapolate values for all 35 time points of treatment, resulting in the final malignant cell proportion vector m_{p4510} corresponding to all treatment samples from patient p4510.

Detection of genes over-expressed in malignant cells. Genes over-expressed in malignant cells were determined using only patient p4510 samples, based on the significance of the fit of a gene's expression values with the malignant cell proportion vector m_{p4510} in the multivariate regression model. The multivariate regression model for the gene expression (ge^i) for a given gene *i* is represented as:

$$\overline{ge^i} = bg^i + \theta_1^i \overline{b_1^i} + \theta_2^i \overline{b_2^i} + \theta_3^i \overline{b_3^i} + \theta_m^i \overline{m_{p4510}},$$

where parameters bg, θ_1, θ_2, θ_3 and θ_m represent the constant background level for the gene, the effect of batch 1, 2 and 3 (b_1, b_2, b_3) on the gene expression and the effect of changes in malignant cell proportion (m_{p4510}) across samples, respectively. Values for patterns b_1, b_2, b_3 were set to 1 for samples belonging to the corresponding batch and 0 otherwise. Pattern m_{p4510} was normalized by the maximum value across used samples. Matlab v 7.2 function *regress* was used for the multivariate linear regression and the significance of the evaluated parameter $\theta_m > 0$ was defined at FDR<5% determined according to the Benjamini-Hochberg procedure across all tested genes. Furthermore, the significant genes were restricted to those whose expression in the final cycles 9-12 was not significantly lower (P>0.5 by one-tail t-test) than the expression of the gene in the 4 healthy donor samples. Genes satisfying these two criteria were considered to be malignant-specific. For every gene, the upregulation fold change (FC) in malignant cells in patient p4510 was calculated as $FC = \frac{\theta_m + bg}{bg}$

Prediction of malignant cell proportion in a sample. In order to develop a malignant cell predictor (MCP) to predict a percentage of malignant cells in a microarray sample, we used a principal component analysis (PCA) approach. The training set consisted of expression data for 1479 genes found to be malignant-specific from 28 treatment time points from patient p4510. A PCA analysis was performed on samples

and we determined projection coefficients for all 28 training samples on the first principal component (P^1_{train}). A double Gaussian curve was used to fit the 28 projection coefficients (x) to a final vector of proportions of malignant cells m_{p4510} determined earlier (R^2 = 0.9937):

$$m_{p4510} = a_1 \cdot e^{-\left(\frac{x-b_1}{c_1}\right)^2} + a_2 \cdot e^{-\left(\frac{x-b_2}{c_2}\right)^2}$$

A projection of a new sample on the P^1_{train} using expression of 1479 genes is used in the fit to predict the proportion of malignant cells in the sample.

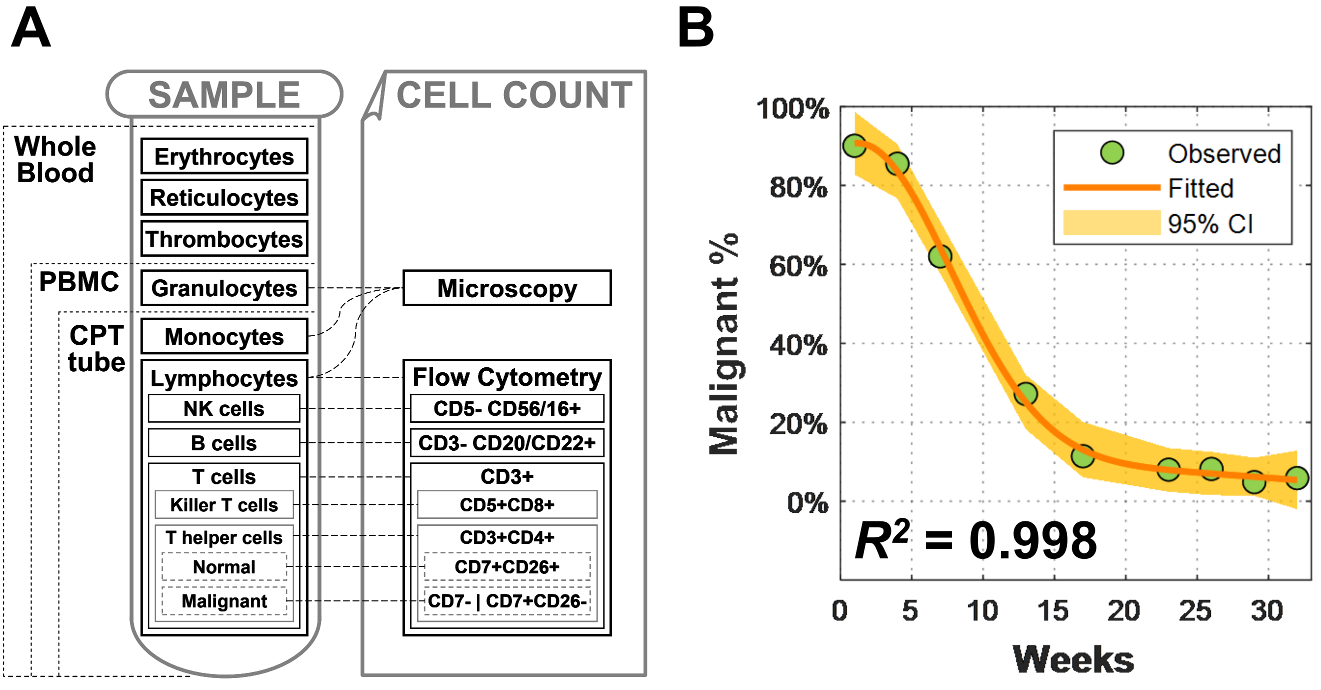

Supplementary Figure S1. Cell populations and malignant cell fraction. A. The figure shows whole blood cell types and their subsets included in the PBMC and CPT tube sample and also indicates the methods used to estimate the numbers of those cell types. **B.** Gaussian fit and 95% confidence interval to the observed malignant cell proportions

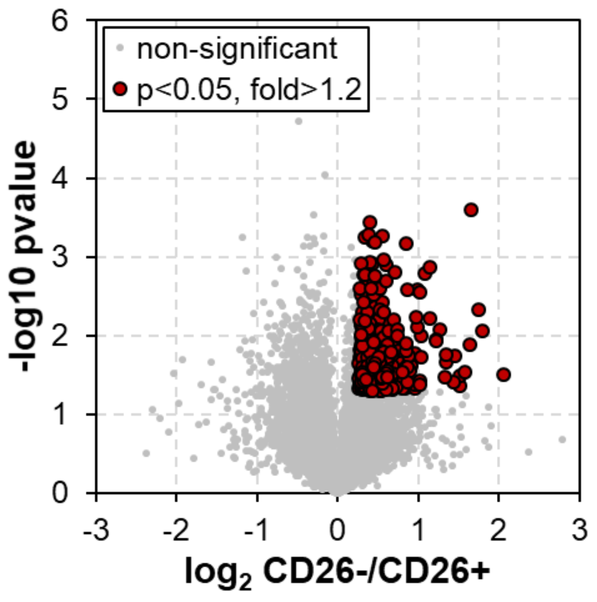

Supplementary Figure S2. Differences between CD3+CD4+CD26- and CD3+CD4+CD26+ cells derived from Sezary Syndrome patients. Red dots (n=334) were considered as an additional evidence for selecting candidates for malignant cell surface markers

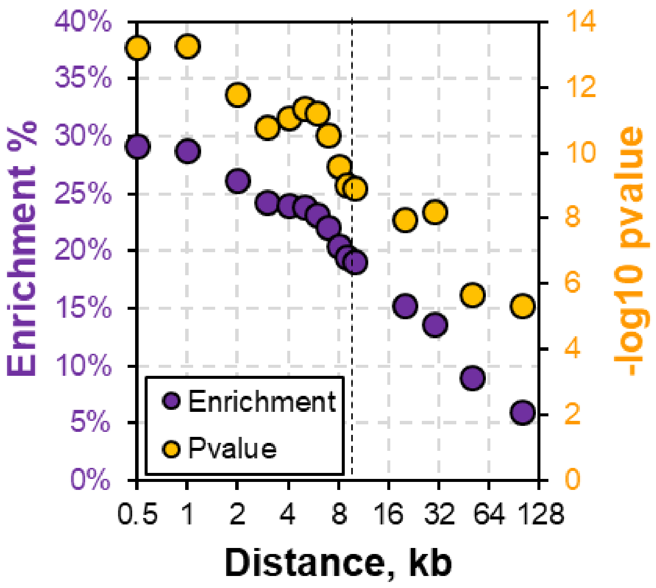

Supplementary Figure S3. Enrichment and its significance of HLF binding sites near MCP genes across varying distance parameter.

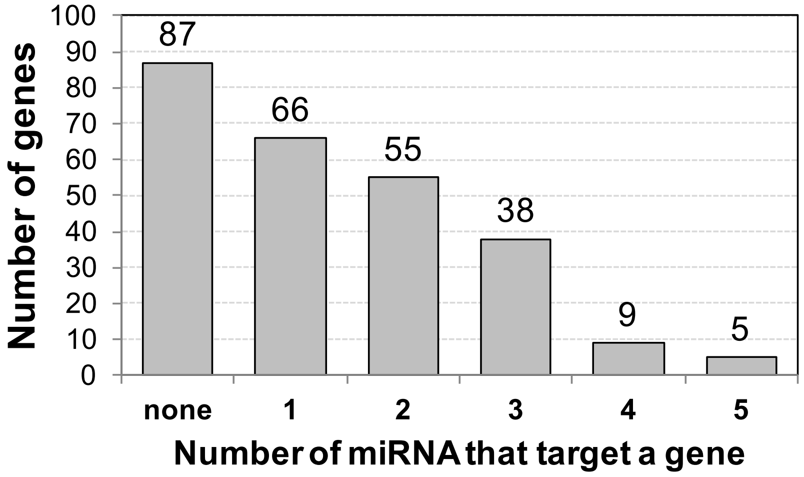

Supplementary Figure S4. Numbers of genes targeted by various numbers of miRNAs.

methylation max vs min	methylation corr rho	methylation mean β	gene
61%	-0.94	40%	GPR15
59%	-0.88	64%	NINJ2
58%	-0.95	65%	GPR81
57%	-0.96	40%	MEOX1
54%	-0.85	38%	LRRN3
53%	-0.76	44%	ICOS
51%	-0.76	35%	IL32
49%	-0.83	43%	PDCD1
47%	-0.89	57%	GPR55
47%	-0.76	66%	CCR8
46%	-0.98	48%	C1orf24
43%	-0.87	31%	ITK
41%	-0.97	84%	FGFR1
40%	-0.75	45%	ARMC2
39%	-0.91	33%	FLJ35801
39%	-0.84	56%	HTR2B
38%	-0.88	20%	FCRL3
38%	-0.94	27%	CD84
38%	-0.86	49%	RAB3IP
37%	-0.83	35%	NPDC1
36%	-0.98	29%	TLOC1
35%	-0.95	25%	LMNA
35%	-0.60	32%	ZFP37
34%	-0.93	28%	AFAP
34%	-0.75	29%	GPR171
34%	-0.93	23%	GCET2
32%	-0.80	17%	TETRAN
32%	-0.96	25%	FLJ11016
32%	-0.90	61%	RASGRP3
32%	-0.96	11%	PGDS
31%	-0.94	26%	ANAPC4
31%	-0.87	61%	GCNT2
31%	-0.94	17%	KIAA1199
30%	-0.77	69%	PCSK1N
28%	-0.82	11%	PTPLA
28%	-0.46	33%	CD82
27%	-0.97	26%	TP73L
25%	-0.79	14%	PLS3
24%	-0.87	35%	CASK
24%	-0.73	46%	KCTD17
23%	-0.74	83%	FAM107B
23%	-0.57	38%	SDCCAG3
23%	-0.84	20%	C1orf71
23%	-0.76	45%	C1orf115
23%	-0.76	38%	HOXA5
22%	-0.86	5%	EEF1A2
22%	-0.60	35%	MLC1SA
21%	-0.71	20%	SCP2
21%	-0.87	48%	KCNQ2
21%	-0.90	29%	OTOF
21%	-0.66	13%	KCNK1
20%	-0.89	62%	NGFRAP1

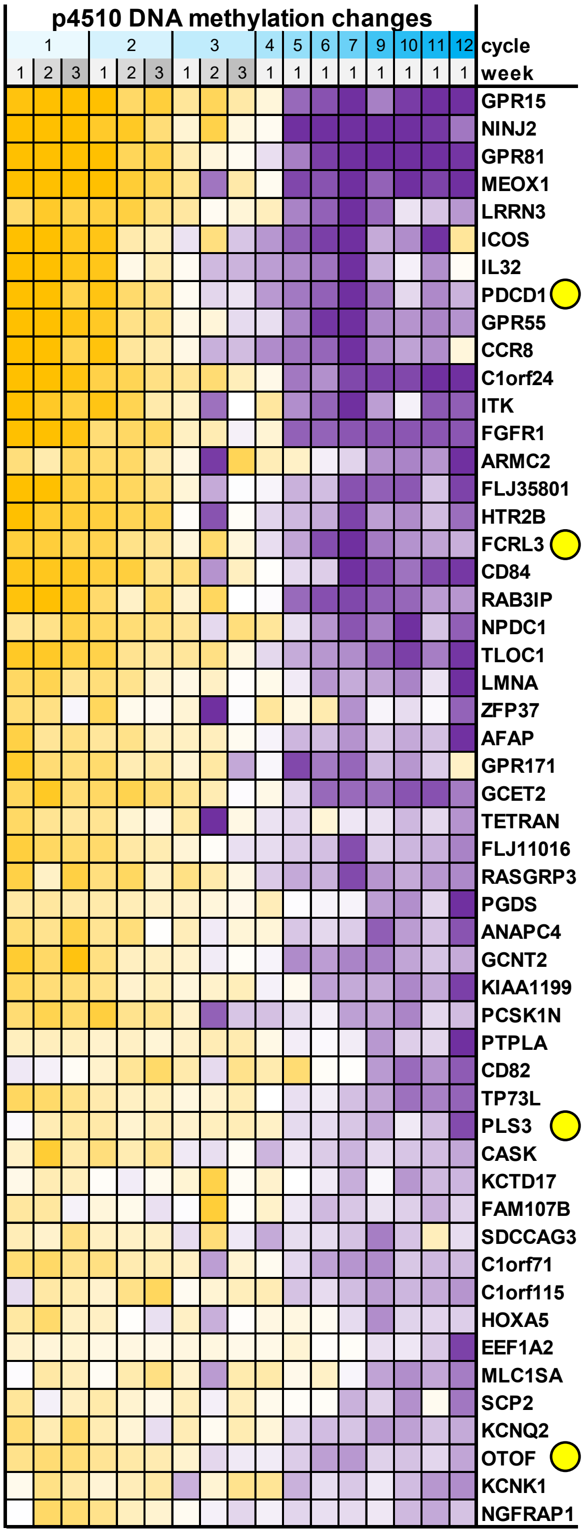

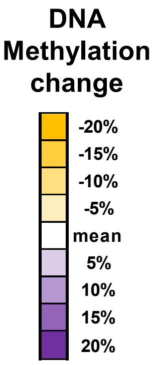

Supplementary Figure S5. Methylation changes among top changed MCP genes. DNA methylation changes for 52 MCP genes changed at least 2 fold across treatment cycles for p4510 are shown. Genes with significant negative correlation vs proportion of malignant cells were filtered using FDR<5%, at least 20% methylation change thresholds. rho = Spearman correlation coefficient. Methylation beta values are shown as % methylated signal. Notable genes discussed in results are highlighted with yellow circles

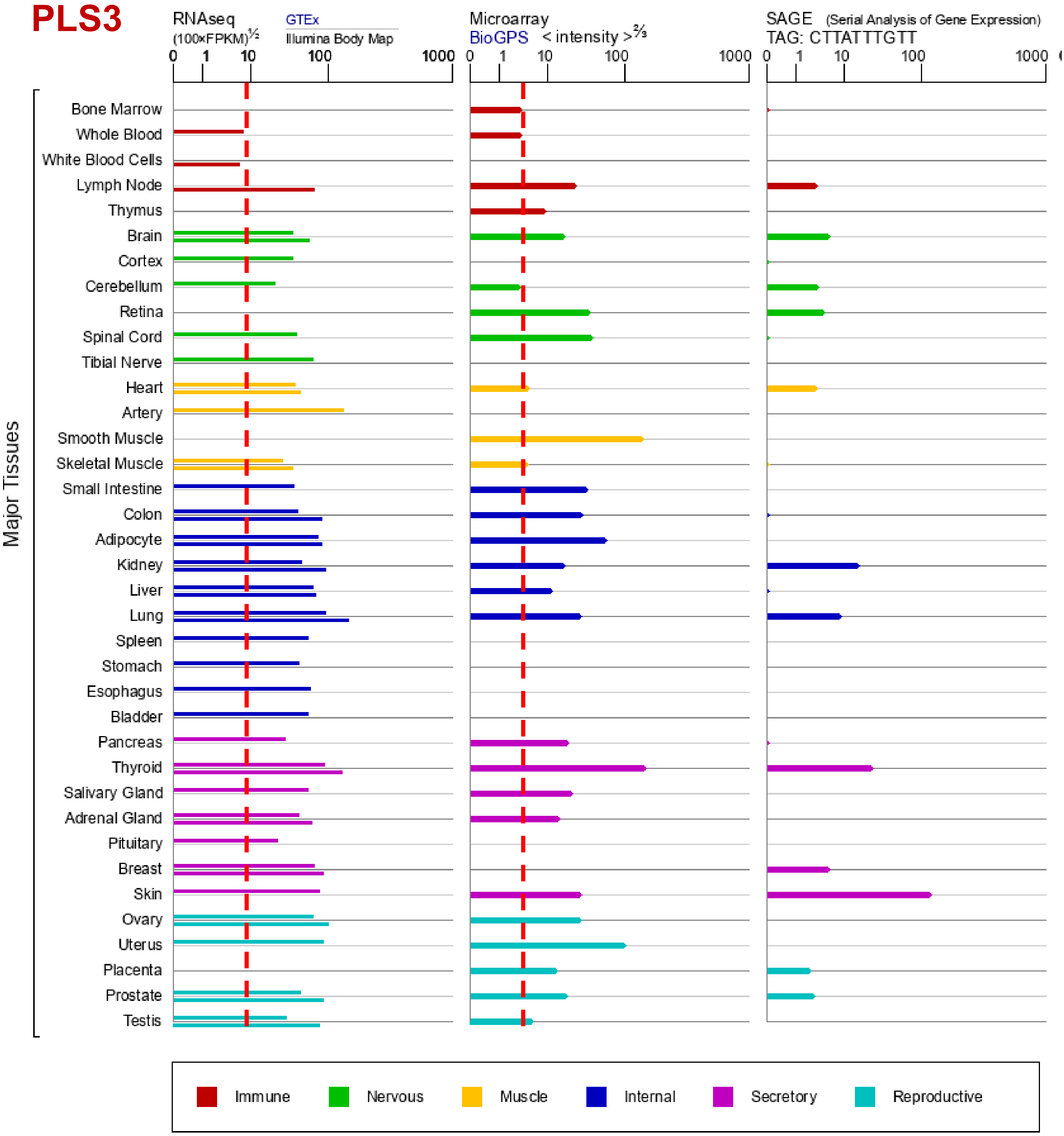

Supplementary Figure S6. Expression of PLS3 mRNA across multiple tissue types as derived from genecards.org. The figure demonstrate lowest expression of PLS3 in blood cells, whole blood or only leukocytes. Red dashed lines serves as a visual indication that expression of PLS3 in blood is at background noise levels compared to any other tissue types.

Source: https://www.genecards.org/cgi-bin/carddisp.pl?gene=PLS3

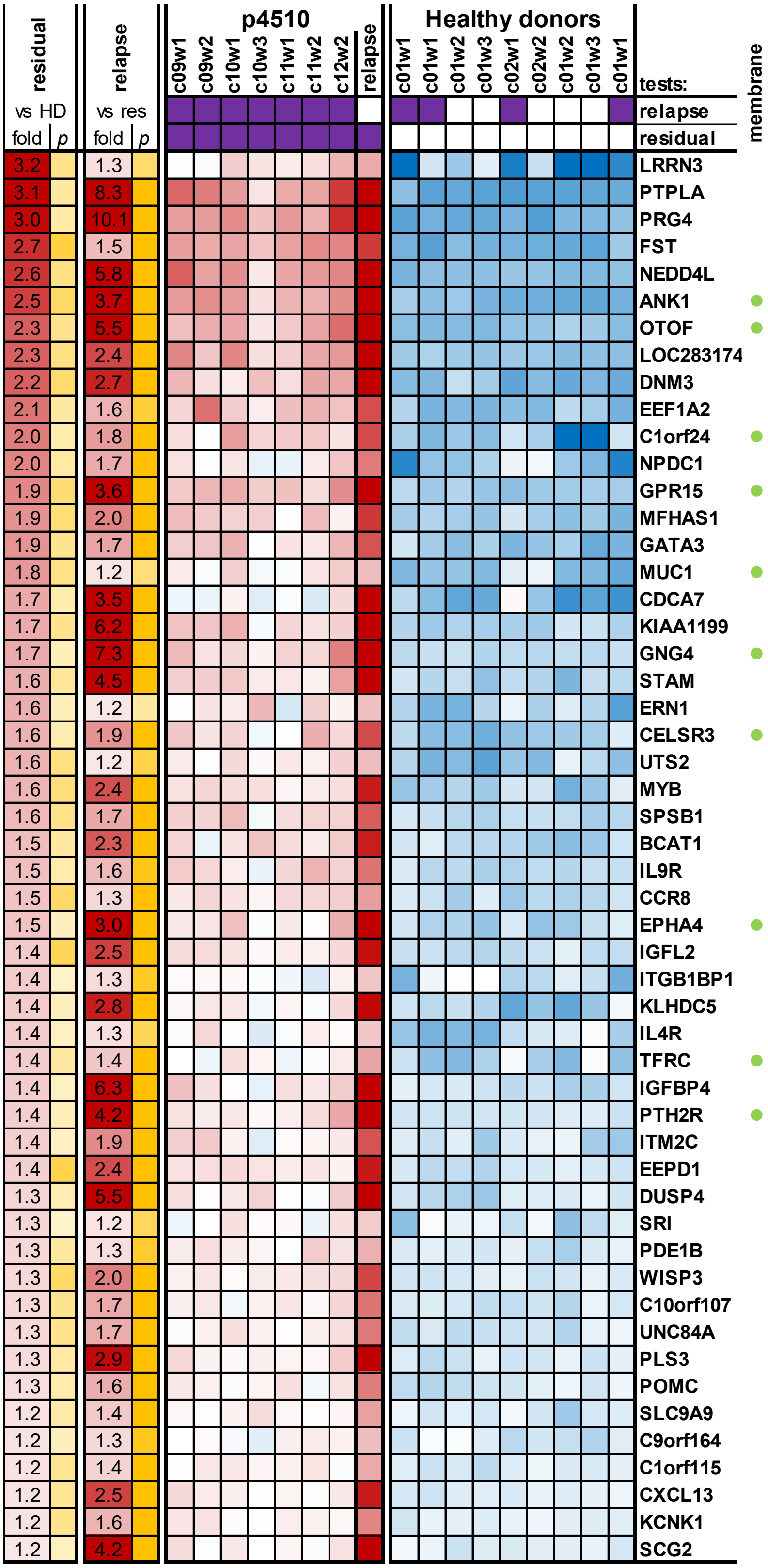

Supplementary Figure S7. Full list of 52 markers associated with residual disease. Expression heatmap of genes detected at a significantly higher levels (FDR<5%, at least 1.2-fold) in remission samples than normal levels of healthy donors (at least 1.2-fold) and showed an increase in the relapse sample after 9 months after therapy (at least 1.2 fold).

Subjects group	Subject ID. time point	% CD4+CD26- T cells	% CD4+CD26- T cells that are:		
			CD164+	PD1+	MUC1+
Sezary Syndrome patients	S01	4.8%	35.9%	31.6%	10.9%
	S02	5.4%	37.1%	65.5%	20.2%
	S03	5.7%	40.6%	38.6%	14.2%
	S04	6.8%	15.2%	19.9%	22.8%
	S05	9.4%	60.4%	44.5%	67.7%
	S06	11.0%	12.7%	37.8%	5.2%
	S07	11.5%	27.3%	44.6%	1.8%
	S08	12.1%	39.2%	38.9%	8.8%
	S09	14.8%	54.4%	42.2%	36.8%
	S10	23.0%	33.1%	25.7%	8.9%
	S11	25.1%	13.6%	35.5%	13.5%
	S12	32.9%	2.9%	3.9%	6.9%
	S13	39.9%	47.5%	85.2%	18.0%
	S14	43.6%	17.6%	70.9%	35.6%
	S15	69.7%	48.9%	37.9%	12.6%
	S16	75.5%	72.5%	32.0%	27.6%
	S17	73.3%	78.6%	82.2%	25.4%
	S18	73.5%	75.2%	75.5%	15.8%
	S19	76.0%	22.3%	19.8%	1.0%
	S20	78.9%	35.9%	40.3%	3.8%
	S21	89.6%	78.6%	1.4%	8.2%
	S22	94.9%	93.8%	11.2%	18.5%
Romidepsin trial patients	p4502.a	11.6%	55.7%	33.0%	16.2%
	p4503.b	77.5%	98.0%	90.0%	7.5%
	p4510.c	78.7%	86.4%	14.8%	5.2%
	p4510.d	91.7%	50.2%	14.1%	15.9%
	p4510.e	8.4%	25.5%	4.6%	9.5%
	p4510.f	5.7%	19.9%	42.2%	na
Healthy donors	HD356	5.6%	14.3%	36.5%	8.7%
	HD330	7.1%	11.6%	12.8%	2.2%
	HD988	5.8%	40.2%	33.2%	5.5%
	HD67	8.9%	23.8%	2.9%	3.5%
	HD970	11.1%	6.8%	23.5%	11.0%
	HD462	14.1%	12.7%	6.8%	na
	HD647	11.9%	16.8%	20.0%	na
	HD238	12.1%	22.0%	27.4%	na

Supplementary Figure S8. Flow cytometry measurements performed across different subjects. Timepoints: a = 3 years after treatment, b = 3 years before treatment, c = 4 years before treatment, d = 2 months before treatment, e = at recurrence (10 months after treatment termination), f = post bone marrow transplant (4 years after treatment termination).

Supplementary Tables

Supplementary Table S1. Types of data that were used in the study for every patient. ge = mRNA microarray gene expression, c = cell count, mi = miRNA microarray expression, me = promoter methylation arrays. Recurrence = recurrence sample taken after 1 year after the end of the treatment.

cycle	week	p4502	p4503		p4507		p4509		p4510			
		ge	ge	c	ge	c	ge	c	ge	c	mi	me
1	1	+	+	+	+	+	+	+	+	+	+	+
	2	+	+	+	+		+		+		+	+
	3	+	+	+	+		+		+		+	+
2	1	+	+		+	+	+	+	+	+	+	+
	2		+		+				+		+	+
	3				+				+		+	+
3	1		+		+	+	+		+	+	+	+
	2		+		+				+		+	+
	3				+				+		+	+
4	1				+	+			+		+	+
	2				+							
	3				+				+			
5	1				+				+	+	+	+
	2				+				+			
	3				+				+			
6	1				+				+		+	+
	2				+	+			+	+		
	3				+							
7	1								+		+	+
	2								+			
	3								+			
8	1								+	+	+	
	2								+			
	3											
9	1								+	+	+	+
	2								+			
	3											
10	1								+	+		+
	2											
	3								+			
11	1								+	+	+	+
	2								+			
	3											
12	1											+
	2								+			
	3											
recurrence									+	+		

Supplementary Table S2. Percent of blood involvement for patients, whose PBMC samples were obtained from NIH for the purpose of independent external validation.

Institute	patient number	Unique ID	% blood involvement
NIH	1	nih.p1	0.0%
NIH	2	nih.p2	0.0%
NIH	3	nih.p3	1.8%
NIH	4	nih.p4	5.4%
NIH	5	nih.p5	7.3%
NIH	6	nih.p6	13.0%
NIH	7	nih.p7	14.4%
NIH	8	nih.p8	16.3%

Supplementary Table S3. Enriched categories in the list of 1479 MCP genes. Term: annotation term enriched, E=enrichment: value of enrichment, Sens=sensitivity: N/K (P%), where N=number of genes with the annotation in the list, K=number of such genes known overall. *P*: Fisher Exact test p-value of the enrichment, FDR=false discovery rate.

Term	E	Sens	*P*	FDR	genes (Malignant/Normal)
GO:0010389: regulation of G2/M transition of mitotic cell cycle	8.1	4/5 (80%)	0.008	13.9%	CDKN2A (1.12), CENPF (1.45), PKIA (3.40), PRKCQ (4.07)
GO:0000780: condensed nuclear chromosome, centromeric region	7.3	5/7 (71%)	0.002	3.6%	BUB1 (1.14), REC8L1 (1.37), SGOL1 (1.23), SGOL2 (1.28), SUV420H1 (1.22)
GO:0046870: cadmium ion binding	7.3	5/7 (71%)	0.003	3.9%	MT1A (1.51), MT1F (1.97), MT1G (1.71), MT1X (1.38), NOS3 (1.14)
PIRSF002564:metallothionein	7.2	5/8 (62%)	0.003	4.3%	MT1A (1.51), MT1F (1.97), MT1G (1.71), MT1X (1.38), MT3 (1.96)
IPR018459:RII binding domain	6.3	5/8 (62%)	0.005	8.0%	AKAP1 (1.09), AKAP12 (1.15), AKAP3 (1.22), MAP2 (1.30), PALM2-AKAP2 (1.09)
GO:0045841: neg regulation of mitotic metaphase/anaphase transition	5.1	5/10 (50%)	0.01	19.9%	BUB1 (1.14), CENPF (1.45), PRG4 (2.00), TERF1 (1.99), TPR (11.37), TTK (1.20)
IPR001936: Ras GTPase-activating protein	5.0	5/10 (50%)	0.01	19.1%	IQGAP3 (3.43), NF1 (1.56), RASA1 (1.23), RASA3 (1.23), RASAL2 (1.24)
GO:0050927: positive regulation of positive chemotaxis	4.3	6/14 (42%)	0.009	14.6%	AGER (1.09), CCR4 (6.98), EDG1 (3.42), ITGA2 (1.74), PRKCA (1.30), SCG2 (2.00)
SP_PIR: metal binding	4.1	7/17 (41%)	0.005	6.9%	ATP7B (3.49), ITGA3 (1.87), ITGA9 (1.18), MT1A (1.51), MT1F (1.97), MT1G (1.71), MT1X (1.38)

SP_PIR: protein kinase inhibitor	4.0	6/15 (40%)	0.01	17.7%	CDKN2A (1.12), CDKN2C (1.19), PKIA (3.40), PKIB (1.12), TRIB2 (5.66), TRIB3 (2.12)
GO:0030071: regulation of mitotic metaphase/anaphase transition	3.9	8/21 (38%)	0.003	5.3%	ANAPC10 (1.42), BUB1 (1.14), CDC23 (1.54), CENPF (1.45), DLG7 (1.25), PRG4 (2.00), TERF1 (1.99), TPR (11.37), TTK (1.20)
GO:0007131: reciprocal meiotic recombination	3.7	7/19 (36%)	0.008	13.5%	LIG3 (1.41), MRE11A (1.26), RAD51C (1.64), RAD51L1 (1.12), RAD51L3 (1.53), REC8L1 (1.37), TRIP13 (1.32)
GO:0004860: protein kinase inhibitor activity	3.1	10/33 (30%)	0.004	5.6%	CASP3 (1.75), CDKN2A (1.12), CDKN2C (1.19), IBTK (3.09), MBIP (1.91), PKIA (3.40), PKIB (1.12), SH3BP5 (1.27), TRIB2 (5.66), TRIB3 (2.12)
GO:0007127: meiosis I	2.9	9/32 (28%)	0.01	17.7%	CKS2 (1.87), LIG3 (1.41), MRE11A (1.26), RAD51C (1.64), RAD51L1 (1.12), RAD51L3 (1.53), REC8L1 (1.37), SYCP2 (1.82), TRIP13 (1.32)
GO:0000794: condensed nuclear chromosome	2.8	11/40 (27%)	0.004	6.0%	BUB1 (1.14), MKI67IP (1.34), REC8L1 (1.37), RRS1 (1.47), SGOL1 (1.23), SGOL2 (1.28), SUV420H1 (1.22), SYCP2 (1.82), TEX12 (1.24), TTN (1.07), TUBG1 (1.48)
UP_SEQ: zinc finger region:C2H2-type	2.7	10/37 (27%)	0.010	16.4%	ARID2 (1.21), C1orf25 (1.23), FOXP2 (1.11), FOXP4 (1.15), KCMF1 (1.24), NUFIP1 (1.11), SCAPER (1.63), TANK (1.31), ZNF608 (1.29), ZNF706 (1.67)
GO:0003690: double-stranded DNA binding	2.3	18/81 (22%)	0.002	3.0%	CGGBP1 (2.07), CREB1 (2.23), HLF (1.39), HNRPDL (1.83), KLF6 (1.60), MEN1 (1.11), MRE11A (1.46), NR5A1 (1.25), PURB (1.78), RAD51AP1 (1.32), RBMS1 (2.88), RECQL (1.47), STAT5B (2.05), TERF1 (1.99), THRB (1.32), TP73L (2.69), ZEB1 (1.24), ZNF148 (1.36)

hsa04120:Ubiquitin mediated proteolysis	2.2	26/127 (20%)	0.0001	0.2%	AIRE (6.70), ANAPC10 (1.42), ANAPC11 (1.49), ANAPC2 (1.12), ANAPC4 (2.33), BIRC3 (9.02), BIRC6 (2.08), CDC23 (1.54), CUL3 (1.50), CUL4A (1.15), CUL4B (1.35), DDB1 (1.36), ERCC8 (1.43), FBXW7 (1.11), MDM2 (1.19), NEDD4 (1.29), NEDD4L (2.00), PML (1.33), SMURF2 (1.11), TCEB1 (2.38), TRIM32 (2.02), TRIM37 (1.95), UBE2B (1.80), UBE2H (1.36), UBE2Z (1.61), UBR5 (2.15)
IPR018029:C2 membrane targeting protein	2.1	15/72 (20%)	0.01	16.3%	CPNE2 (2.99), KIAA0528 (2.23), MCTP1 (2.16), NEDD4 (1.29), NEDD4L (2.00), OTOF (2.00), PIK3C2B (1.18), PLCE1 (1.08), PLCG1 (2.50), PLCL2 (2.28), PRKCA (1.30), RAB11FIP5 (1.40), RASA1 (1.23), RASA3 (1.23), SMURF2 (1.11)
GO:0006469: negative regulation of protein kinase activity	2.1	16/78 (20%)	0.008	14.3%	CASP3 (1.75), CAV1 (1.11), CDKN2A (1.12), CRIPAK (1.37), DUSP9 (1.08), MBIP (1.91), MEN1 (1.11), NF1 (1.56), NUP62 (1.49), PAK2 (3.36), PKIA (3.40), PKIB (1.12), PRKCA (1.30), SH3BP5 (1.27), SPRY2 (3.22), TRIB3 (2.12)
GO:0051321: meiotic cell cycle	2.1	16/79 (20%)	0.010	15.9%	BOLL (1.27), CDC25B (1.42), CKS2 (1.87), LIG3 (1.41), MRE11A (1.46), RAD51C (1.64), RAD51L1 (1.12), RAD51L3 (1.53), REC8L1 (1.37), SGOL1 (1.23), SGOL2 (1.28), SYCP2 (1.82), TAF1L (1.49), TRIP13 (1.32), TUBG1 (1.48), UTP14C (3.23)
GO:0051348: negative regulation of transferase activity	2.0	17/85 (20%)	0.008	13.9%	CASP3 (1.75), CAV1 (1.11), CDKN2A (1.12), CRIPAK (1.37), DUSP9 (1.08), MBIP (1.91), MEN1 (1.11), NF1 (1.56), NUP62 (1.49), PAK2 (3.36), PKIA (3.40), PKIB (1.12), PRKCA (1.30), SH3BP5 (1.27), SPRY2 (3.22), TERF1 (1.99), TRIB3 (2.12)

UP_SEQ: zinc finger region:C2H2-type 1; degenerate	2.0	18/89 (20%)	0.007	12.3%	FLJ16231 (1.44), LOC115648 (1.23), LOC91661 (1.12), ZNF121 (2.39), ZNF136 (1.36), ZNF233 (1.30), ZNF234 (1.75), ZNF235 (1.31), ZNF273 (1.31), ZNF30 (2.56), ZNF347 (1.36), ZNF416 (1.27), ZNF429 (1.91), ZNF493 (1.19), ZNF559 (1.96), ZNF577 (1.32), ZNF625 (1.11), ZNF818 (1.34), ZNF93 (1.21)
GO:0043566: structure-specific DNA binding	2.0	25/127 (19%)	0.001	2.0%	CGGBP1 (2.07), CREB1 (2.23), HLF (1.39), HNRPDL (1.83), HSPD1 (1.41), KLF6 (2.25), MCM4 (1.33), MCM6 (1.21), MEN1 (1.11), MRE11A (1.46), NR5A1 (1.25), POLG2 (1.76), PURB (1.78), RAD51AP1 (1.32), RBMS1 (2.88), RECQL (1.47), RPA3 (1.49), STAT5B (2.05), TERF1 (1.99), THRB (1.32), TP73L (2.69), WBP11 (1.18), WRN (1.93), ZEB1 (1.24), ZNF148 (1.36)

Supplementary Table S4. miRNAs that were significantly anti-correlated with malignant cell counts and show enrichment of predicted targets in the list of malignant-specific genes.

**Variable**		**All 6 miRNAs**	let-7	mir-10	mir-154	mir-30	mir-329	mir-548
miRNA fold change After/before treatment		>2	3.02	4.34	2.65	10.16	5.11	2.30
Spearman correlation with malignant cell count, pvalue		<0.001	$5x10^{-5}$	0	0.0009	0.0008	0.0006	0
in 260 gene list	Predicted targets	173	24	9	107	33	50	128
	Not predicted	87	236	251	153	227	210	132
not in 260 gene list	Predicted target	6429	706	173	3712	1038	1629	4308
	Not predicted	6108	11831	12364	8825	11499	10908	8229
% of malignant-specific that may be targeted		67%	9%	3%	41%	13%	19%	49%
% of NOT-malignant-specific that may be targeted		51%	6%	1%	30%	8%	13%	34%
Enrichment		1.30	1.64	2.51	1.39	1.53	1.48	1.43
Fisher p-value		10^{-6}	0.020	0.012	$9x10^{-5}$	0.017	0.005	10^{-6}
